# Investigating the effects of long-term Aroclor 1260 exposure on fatty liver disease in a diet-induced obesity mouse model

**DOI:** 10.3389/fgstr.2023.1180712

**Published:** 2023-05-12

**Authors:** Kimberly Z. Head, Oluwanifemi E. Bolatimi, Tyler C. Gripshover, Min Tan, Yan Li, Timothy N. Audam, Steven P. Jones, Carolyn M. Klinge, Matthew C. Cave, Banrida Wahlang

**Affiliations:** 1 Division of Gastroenterology, Hepatology, and Nutrition, Department of Medicine, School of Medicine, University of Louisville, Louisville, KY, United States; 2 The Hepatobiology and Toxicology Center, University of Louisville, Louisville, KY, United States; 3 Department of Pharmacology and Toxicology, School of Medicine, University of Louisville, Louisville, KY, United States; 4 Department of Surgery, School of Medicine, University of Louisville, Louisville, KY, United States; 5 Center for Cardiometabolic Science, Department of Medicine, Division of Environmental Medicine, Christina Lee Brown Envirome Institute, University of Louisville, Louisville, KY, United States; 6 Department of Biochemistry and Molecular Genetics, School of Medicine, University of Louisville, Louisville, KY, United States; 7 The Center for Integrative Environmental Health Sciences, University of Louisville, Louisville, KY, United States; 8 University of Louisville (UofL) Superfund Research Center, University of Louisville, Louisville, KY, United States; 9 Robley Rex Department of Veterans Affairs Medical Center, Louisville, KY, United States

**Keywords:** Aroclor 1260, PCBs, NAFLD, TAFLD, steatohepatitis, hepatocellular carcinoma

## Abstract

**Introduction:**

Polychlorinated biphenyls (PCBs) are persistent environmental toxicants that have been implicated in numerous health disorders including liver diseases such as non-alcoholic fatty liver disease (NAFLD). Toxicant-associated NAFLD, also known as toxicant-associated fatty liver disease (TAFLD), consists of a spectrum of disorders ranging from steatosis and steatohepatitis to fibrosis and hepatocellular carcinoma. Previously, our group demonstrated that 12-week exposure to the PCB mixture, Aroclor 1260, exacerbated steatohepatitis in high-fat diet (HFD)-fed mice; however, the longer-term effects of PCBs on TAFLD remain to be elucidated. This study aims to examine the longer-term effects of Aroclor 1260 (>30 weeks) in a diet-induced obesity model to better understand how duration of exposure can impact TAFLD.

**Methods:**

Male C57BL/6 mice were exposed to Aroclor 1260 (20 mg/kg) or vehicle control by oral gavage at the beginning of the study period and fed either a low-fat diet (LFD) or HFD throughout the study period.

**Results:**

Aroclor 1260 exposure (>30 weeks) led to steatohepatitis only in LFD-fed mice. Several Aroclor 1260 exposed LFD-fed mice also developed hepatocellular carcinoma (25%), which was absent in HFD-fed mice. The LFD+Aroclor1260 group also exhibited decreased hepatic *Cyp7a1* expression and increased pro-fibrotic *Acta2* expression. In contrast, longer term Aroclor 1260 exposure in conjunction with HFD did not exacerbate steatosis or inflammatory responses beyond those observed with HFD alone. Further, hepatic xenobiotic receptor activation by Aroclor 1260 was absent at 31 weeks post exposure, suggesting PCB redistribution to the adipose and other extra-hepatic tissues with time.

**Discussion:**

Overall, the results demonstrated that longer-term PCB exposure worsened TAFLD outcomes independent of HFD feeding and suggests altered energy metabolism as a potential mechanism fueling PCB mediated toxicity without dietary insult. Additional research exploring mechanisms for these longer-term PCB mediated toxicity in TAFLD is warranted.

## Introduction

Polychlorinated biphenyls (PCBs) are environmental toxicants associated with numerous health effects such as cardiovascular complications and metabolic diseases in exposed populations (1–3). Categorized as persistent organic pollutants (POPs), PCBs continue to threaten public health due to their resistance to degradation and bioaccumulation in living organisms (4). PCBs were initially manufactured for commercial use primarily as dielectric fluids in electrical capacitors and transformers, in addition to other industrial applications and uses (5). Due to their carcinogenic properties, PCB production and use was halted by the US Congress in 1979, and worldwide at the historic Stockholm Convention on POPs in 2001. Despite being banned decades ago, PCBs continue to be detected in the air and water (6, 7). Moreover, appreciable levels of PCBs continue to be detected in the general American adult population as reported in the US National Health and Nutrition Examination Surveys (NHANES) (8). Human exposures to PCBs today occur primarily through ingestion of PCB-contaminated food and water, particularly ocean-based food sources (9, 10).

Depending on the number of chlorine atom substitutions in the biphenyl ring, there are up to 209 individual PCB congeners possible (4). Approximately 130 of these congeners were commercially produced and marketed as mixtures of PCBs. In North America, PCBs were predominantly produced by Monsanto Corporation at its industrial production plant situated in Anniston, Alabama. Monsanto manufactured and sold PCB mixtures under the brand name ‘Aroclor’ with Aroclor 1260 being one of Monsanto’s first generation Aroclors marketed during the 1920s (4, 5, 11). Aroclor 1260 contains 60% chlorine by weight, rendering it highly toxic, and was later replaced by lesser chlorinated Aroclors including Aroclor 1016. Nevertheless, due to its high chlorination, Aroclor 1260 is resistant to metabolism, leading to bioaccumulation in living organisms (4, 12). Structurally, based on the position of chlorine atoms in the biphenyl ring, PCB congeners can be classified as either coplanar or non-coplanar. Coplanar PCBs have either one or no chlorine atom substitution in the *ortho* position of the biphenyl ring and are generally lower molecular weight congeners (e.g., PCBs 77 and 126) (12). Coplanar PCBs tend to have a higher binding affinity to the aryl hydrocarbon receptor (AhR), similar to dioxin and may also be termed ‘dioxin-like’ PCBs (13). In contrast, non-coplanar PCBs are congeners with more than one *ortho*-substituted chlorine atoms in the biphenyl ring, which tend to be higher molecular weight congeners that are more heavily chlorinated and resistant to metabolism (e.g., PCB 153) (12). Non-coplanar PCBs can bind and activate the constitutive androstane receptor (CAR), like phenobarbital, and are also known as ‘phenobarbital-like’ or ‘non-dioxin-like’ PCBs. Because of its high chlorine content and constitution of primarily higher molecular weight congeners that are not readily metabolized, Aroclor 1260 is often considered a non-dioxin-like PCB mixture (11). Importantly, the PCB congener composition in Aroclor 1260 strongly resembles PCB bioaccumulation patterns in humans thus reflecting relevance to current PCB exposures (12, 14).

PCB contribution to the development of metabolic diseases, including non-alcoholic fatty liver disease (NAFLD) has been well-documented (15). NAFLD is a spectrum of pathologic disorders in the liver initially characterized by steatosis or fat accumulation in the hepatocytes. Steatosis can be accompanied by inflammation leading to steatohepatitis which can further progress to fibrosis and/or cirrhosis. The observed NAFLD and its more severe form non-alcoholic steatohepatitis (NASH), when associated with environmental chemical exposures, are often termed as toxicant-associated fatty liver disease (TAFLD) and toxicant associated steatohepatitis (TASH) respectively (16). Positive associations between PCB levels and liver injury markers including elevated liver enzymes and proinflammatory cytokines have been reported in human epidemiologic studies (15). Moreover, both dioxin-like PCBs and non-dioxin-like PCBs have been demonstrated to cause steatosis, hepatic inflammation and injury, and disruption of overall energy metabolism in different experimental laboratory models (15). Additionally, both PCBs and NAFLD (17) have both been associated with the development of cardiovascular diseases, implicating the importance of investigating PCB effects on cardiometabolic outcomes as well. Previous studies from our group have shown that exposure to Aroclor 1260 at environmentally relevant doses for 12-weeks exacerbated steatohepatitis, worsened systemic inflammation, and caused metabolic disruption in a diet-induced obesity (DIO) mouse model, partially through hepatic receptor activation including CAR and AHR, and PCB-diet interactions (11). Further analyses of the hepatic proteome and phospho-proteome in this model revealed that the PCB-induced alterations in the liver also led to early-stage fibrosis and secondary necrosis. Identified mechanisms included hepatic stellate cell activation by PCBs mediated through tumor growth factor beta (TGFβ); and modified oxidative stress responses through PCB-regulated attenuation of nuclear factor erythroid 2-related factor 2 (NRF2) activation (18).

Collectively, these findings suggest that extending the duration of exposure to Aroclor 1260 from 12 weeks to a longer time period in the DIO model should result in established hepatic fibrosis. Furthermore, there are currently no studies that have evaluated the effects of longer-term exposures to PCBs in the context of TAFLD and TASH. Importantly, PCB exposures in humans occur throughout one’s lifespan and can elicit life-long effects, given the nature of bioaccumulation and long half-lives of these ‘forever’ chemicals. Therefore, investigations of PCBs impact on the liver and other PCB target organs with low-dose, longer-term exposures are warranted and are of significance when exploring human PCB exposure paradigms. Thus, the objective of the current study is to evaluate the effects of long-term (>30 weeks) exposures, namely Aroclor 1260, in conjunction with high-fat diet (HFD) feeding to better understand if *i)* longer-term PCB exposure alone is sufficient to induce TAFLD/TASH in the absence of high-fat diets, and if *ii)* longer-term PCB exposure and concurrent high-fat diet feeding will result in exacerbated fibrosis. In addition, *iii)* the current study also assessed longer-term PCB effects on cardiovascular endpoints as a secondary endpoint. The findings from this study will provide insight on the impact of long-term exposures to POPs including PCBs on liver and metabolic health.

## Materials and methods

### Animal studies

**FIGURE 1:**
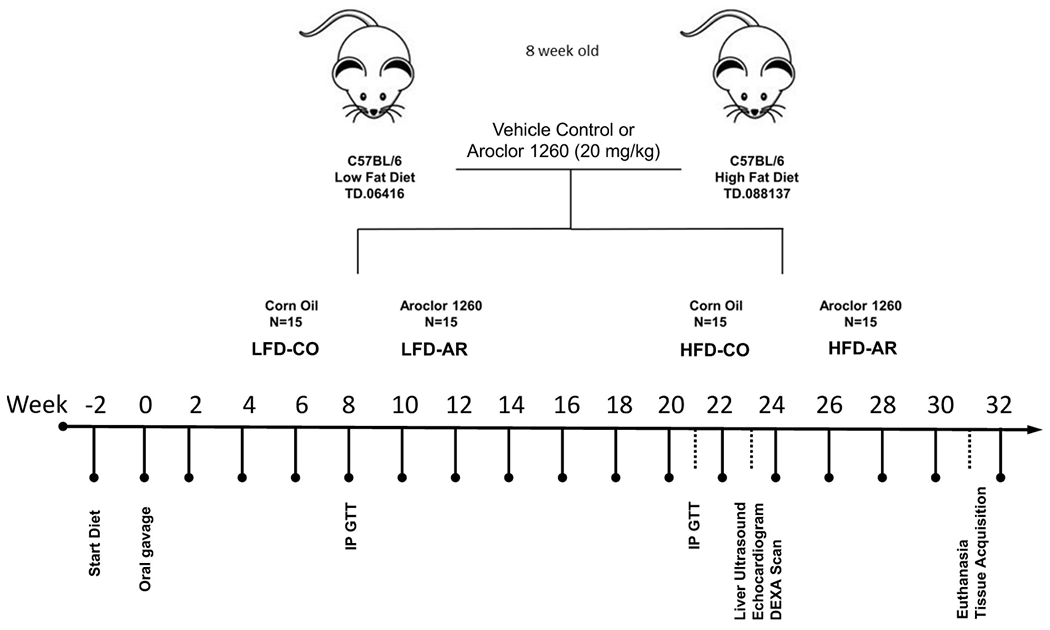
Schematic diagram of the study timeline with respect to HFD feeding, Aroclor 1260 administration and additional experimental procedures.

The current study was approved by the University of Louisville Institutional Animal Care and Use Committee. 8-week-old male C57BL/6J (stock# 0664) mice were purchased from Jackson Laboratory (Bar Harbor, ME, USA). All animals were housed in a temperature and light controlled (12 h light; 12 h dark) room with food and water *ad libitum*. Mice were divided into 4 groups (n=15) and designated with the following abbreviations representing exposure groups as follows: low fat diet + corn oil (LFD-CO), low fat diet + Aroclor 1260 (LFD-AR), high fat diet + corn oil (HFD-CO), and high fat diet + Aroclor 1260 (HFD-AR). A timeline of the animal exposure study and related procedures are illustrated in Figure 1. Mice were allowed to acclimate for two weeks prior to introduction of diet. LFD groups received 10% kcal from fat through a synthetic diet, Teklad, TD.06416 (Envigo, Indianapolis, IN, USA) while high fat diet groups received 42% kcal from fat through a synthetic diet, Teklad TD.88137 (Envigo, Indianapolis, IN, USA). Description of the diets are provided in Supplemental Table 1. Mice were administered either Aroclor 1260 (20 mg/kg) (catalog: C-260N-1G, AccuStandard, New Haven, CT, USA) solubilized in corn oil or vehicle control (corn oil only). Food and body weights were collected biweekly throughout the 31-week study period. A glucose tolerance test was performed at week 8 and week 21 post exposure. Echocardiograms and liver echogenicity *via* ultrasound were assessed at week 23 post exposure. Dual energy x-ray absorptiometry (DEXA) Lunar PIXImus Densitometer (GE Medical Systems, LUNAR, Madison, WI) was used to assess fat and lean tissue mass at 23 weeks post PCB exposure. Mice were euthanized and tissue and plasma samples collected at week 31 post exposure. Final tissue weights were recorded after collection and used to calculate organ to body weight ratios. All tissues were snap frozen in liquid nitrogen and immediately transferred to −80°C for long term storage. Blood was collected from inferior vena cava and anticoagulated with EDTA. Plasma was prepared by centrifugation at 2000g for 10 minutes at 4°C.

### Echocardiograms and liver echogenicity

Transthoracic echocardiography of the left ventricle was performed with the Vevo 3100 High Resolution (Fujifilm VisualSonics, Toronto, ON, Canada) *in vivo* Imaging System, as described for the heart (19, 20) and liver (21). Mice were initially anesthetized with 2% isoflurane with maintenance at 1.5% isoflurane during the examination procedure. Body temperature was maintained at 36.5 −37.5°C using a rectal thermometer interfaced with a controlled heat lamp. Depilatory cream was applied to the mouse’s chest and wiped clean to remove all hair around area of interest. The Vevo MX550D (40 MHz) scan head was used to obtain two-dimensional images of the parasternal long axis loops of the heart. M-modes were taken from the same images. The probe was then adjusted to view the liver, aorta, and mitral valve. Doppler readings of the aorta were obtained as well as still frames and loops of the liver. A standardized region of interest was applied to still images captured from liver ultrasound and correlative mean pixel count data obtained.

### Glucose tolerance test and homeostasis model assessments

Glucose tolerance testing was performed at week 8 and week 21 post Aroclor 1260 oral gavage. Mice were fasted for 6 hours prior to baseline blood sugar assessment. Blood glucose was measured with a handheld glucometer (Contour Next EZ, Ascensia Diabetes Care US, Parsippany, NJ, USA). Mice were challenged with glucose (1 mg/gram body weight) *via* intraperitoneal administration. Glucose levels were measured at 15, 30-, 60-, 90- and 120-minutes post glucose challenge. Results were graphed as glucose concentration in mg/dL versus time with area under the curve (AUC) calculated using the Trapezoid Rule (22). Plasma insulin values were measured using a Rat/Mouse Insulin ELISA assay (EMD Millipore, Burlington, MA, USA). Concentration for unknown samples was extrapolated from a 5-parameter logistic curve generated from absorbance at 450 nm less absorbance at 590 nm of rat insulin standards. Using fasting blood glucose values and insulin values, insulin resistance and beta cell function were assessed. Homeostatic Model Assessment for Insulin Resistance (HOMA-IR) and Homeostasis Model Assessment of Beta Cell Function (HOMA-β) were used as previously described (23). Quantitative insulin sensitivity check index (QUICKI) was also determined using standard mathematical transformations of fasting blood glucose and plasma insulin concentrations (24).

### Histology

Tissues were collected and fixed in 10% neutral buffered formalin for 72 hours before embedding in paraffin wax or in OCT for frozen sections. Oil Red O staining was used to stain 5-micron frozen sections prepared using a cryostat machine. Hematoxylin and eosin (H&E) staining of 5-micron sections from formalin fixed, and paraffin embedded hepatic tissues were performed to detect alterations in morphology and structure. Tissues were heat set at 55°C for 30 minutes to prevent shredding or tissue loss and sequentially cleared through multiple changes of Citrisolv Hybrid (Decon Laboratories, Inc. King of Prussia, PA, USA), then dehydrated by sequential graded alcohol steps, staining with Modified Mayer’s Hematoxylin (Fisher Scientific, Waltham, MA, USA) and counterstained with Alcoholic Eosin Y with phloxine (Millipore Sigma, St. Louis, MO, USA). Hydrated slides were then cover slipped with permanent mounting media and imaged with an Olympus DP74 microscope and CellSens Standard v2.3 software. A second series of slides were stained with saturated picric acid containing 0.1% Sirius Red (Direct Red 80, Millipore Sigma, St. Louis, MO, USA) stain for 30 minutes to visualize collagen bundle staining (25).

### NAFLD activity scoring

According to the previous report by the Pathology Subcommittee of the NASH Clinical Research Network, NAFLD Activity Score (NAS) was analyzed in each H&E-stained slide. NAS was calculated by the sum of scores for steatosis (0–3), lobular inflammation (0–3), and hepatocyte ballooning (0–2). Five fields of view per hepatic sample were scored separately for steatosis, inflammation, and ballooning of hepatocytes as follows: Steatosis: 0, <5%; 1, 5-33%; 2, >33%; 3, > 66%. Lobular Inflammation: 0, no foci; 1/200X, <2 foci/200X; 2, 2-4 foci/200X; 3,>4 foci/200X. Hepatocyte Ballooning: 0, no balloon cells/200X; 1, 1-5 balloon cells/200X; 2,>5 balloon cells/200X (26).

### Plasma adipocytokine and lipid measurements

Plasma levels for alanine aminotransferase (ALT) and aspartate aminotransferase (AST) and circulating levels of high-density lipoprotein (HDL), triglycerides, and cholesterol were obtained using an Abaxis Piccolo Xpress analyzer (Abbott; Abbott Park, IL, USA) with Lipid Panel Plus diskettes (catalog:400-0030; Abaxis Inc.; Union City, CA, USA). Circulating levels of plasma adipokines and cytokines were measured using a MILLIPLEX^®^ Mouse Adipokine Magnetic Bead Panel (Millipore Sigma, Burlington, MA, USA) for analytes interleukin 6 (IL-6), leptin, total plasminogen activator inhibitor 1 (PAI-1), tumor necrosis factor alpha (TNFα), and resistin. Data was obtained from a Luminex 100/200 System equipped with xPONENT Software (Luminex Corporate, Austin, TX, USA). Hepatic lipids were extracted using a modified Bligh and Dyer method with a 2:1 ratio of chloroform to methanol mixture (27, 28). Extracted lipids were measured using Infinity Assay Reagents: Infinity Triglycerides reagent (TR22421, ThermoFisher Scientific, Middletown, VA, USA) and Infinity Cholesterol reagent (TR13421, ThermoFisher Scientific, Middletown, VA, USA). Sample absorbance at 500 nm was calculated from a standard curve constructed from clinical standards (catalog: T7531-STD, C7509-STD; Point Scientific; Canton, MI) for each analyte ran simultaneously with unknown samples.

### RT-PCR

Tissues suspended in RNA Stat 60 (Tel-Test, Inc., Friendswood, TX, USA) were homogenized with 0.5 mm glass beads (Biospec Products, Bartletsville, OK, USA) using a Mini-Beadbeater 16 bead mill homogenizer (Biospec Products, Bartlesville, OK, USA) for 30 seconds to disrupt tissues. Ribonucleic acid (RNA) was extracted from cleared homogenate by chloroform addition and then precipitated by addition of isopropanol. Pelleted RNA was washed with 75% ethanol, evaporated to dryness, and then resolubilized in RNA free diethyl pyrocarbonate (DEPC) treated water. Quality and quantity of RNA was determined using OD 260/280 values obtained from Nanodrop-OneC Microvolume UV-Vis Spectrophotometer (Thermo Fisher). qScript cDNA SuperMix (QuantaBio, Beverly, MA, USA) facilitated first strand cDNA synthesis of 1 μg RNA for Real-Time Polymerase Chain Reaction (RT-PCR). RT-PCR was performed using the Biorad CFX384 TM Real-Time System (Biorad, Hercules, CA USA) using iTaq Universal Probes Supermix (Biorad, Hercules, CA, USA) and selected Taqman probes (Thermo Fisher) as described in Supplemental Table 2. The final RT-PCR volume was 10.5 μL (using iTaq Universal Probes Supermix=5 μL, Taqman probes=0.5 μL each, cDNA=1.5 μL and water=3 μL). Gene expression levels were calculated using the 2-^ΔΔ^Ct method (29–31). Messenger RNA (mRNA) levels were normalized relative to the housekeeping gene (GAPDH) and expressed as fold change relative to the LFD-CO control group, which was set as 1.

### Statistics

All data were analyzed using GraphPad Prism version 9. Two-way ANOVA analysis was performed to determine the effects of ‘diet’ and ‘exposure’ and an interaction effect between diet and exposure. Multiple comparison testing using Tukey’s *post-hoc* test with *p*-value adjustment was performed for group comparisons. For all analysis, significance was set at *p* <0.05 with a 95% confidence level (32). In the event of high data variability in a group, an outlier test was performed using the ROUT method with false discovery rate (FDR) set at 0.1% (33). Lists of *p*-values generated from the statistical analyses for all experimental endpoints are provided in Supplemental Tables 3–8.

## Results

### Effects of Aroclor 1260 and HFD on body composition and food consumption

**FIGURE 2:**
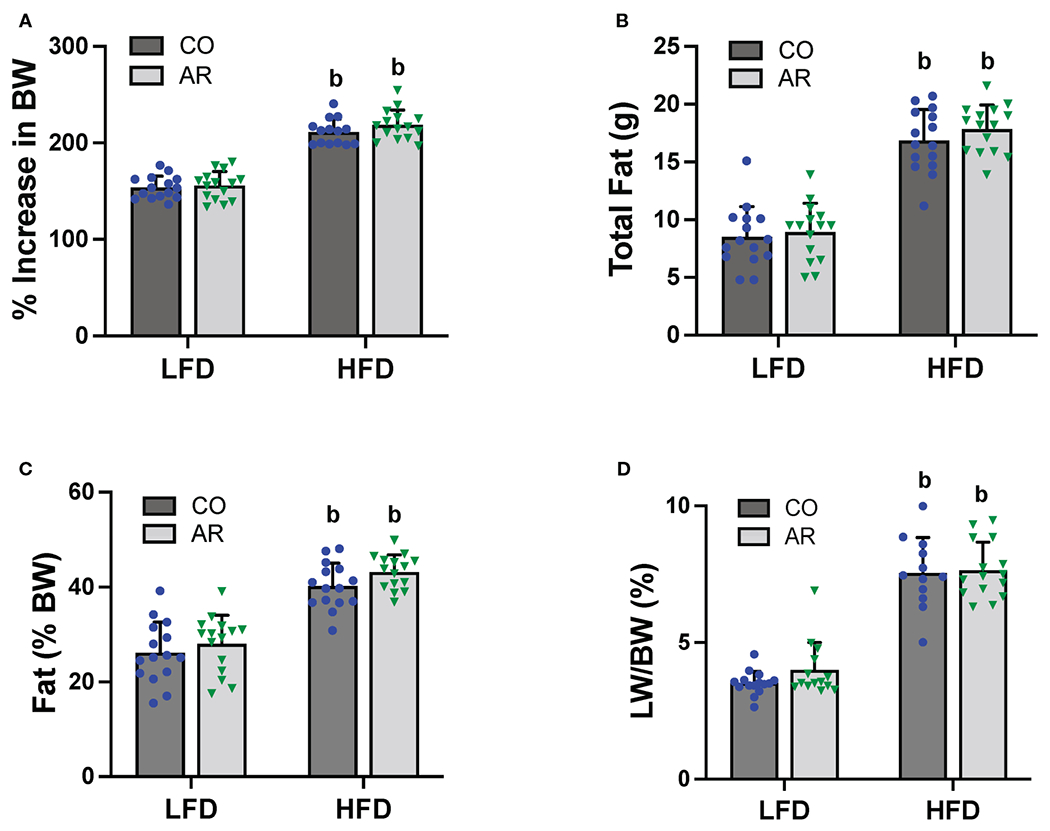
**(A)** Body weight was measured biweekly throughout the study period and percent increase or decrease in body weight (BW) relative to the initial body weight taken at the beginning of the study (considered as 100%) was calculated. Body composition was measured at week 23 of the study period using DEXA scan to obtain **(B)** total fat mass and **(C)** % fat composition was calculated using fat tissue weight obtained from DEXA scanning. **(D)** Livers were isolated and weighed at the end of the study period and liver weight (LW) to body weight ratios were calculated. Values are mean ± SD; *p* <0.05, a - Aroclor 1260 exposure effect, b - diet effect, c - interaction between exposure and diet.

Food consumption and body weights of study animals were collected initially and continued biweekly throughout the study period. Similar to our previous reports (11), HFD-fed mice exhibited a significant increase in body weight gain compared to LFD-fed mice while Aroclor 1260 had no effect in either diet group (Figure 2A and Supplemental Figure 1A). Likewise, HFD-fed mice also showed significantly higher food consumption per mouse per day compared to LFD-fed mice (Supplemental Figure 1B). Body composition including fat and lean mass was measured using DEXA scan on week 23 of the study period. As expected, HFD-fed mice had significantly higher total fat mass and percent body fat (Figures 2B, C), in addition to higher lean tissue mass (Supplemental Figure 1C) compared to LFD-fed mice. HFD-fed mice also exhibited higher white adipose tissue to body weight ratio at the end of the study period as observed by weighing epididymal fat pads obtained post euthanasia (Supplemental Figure 1D). Further, assessment of liver weights measured post euthanasia also demonstrated that HFD feeding increase liver to body weight ratios (Figure 2D) while there was no Aroclor 1260 effect on any of the body composition parameters measured.

### Effects of Aroclor 1260 exposure and HFD on hepatic steatosis, inflammation, and injury

**FIGURE 3:**
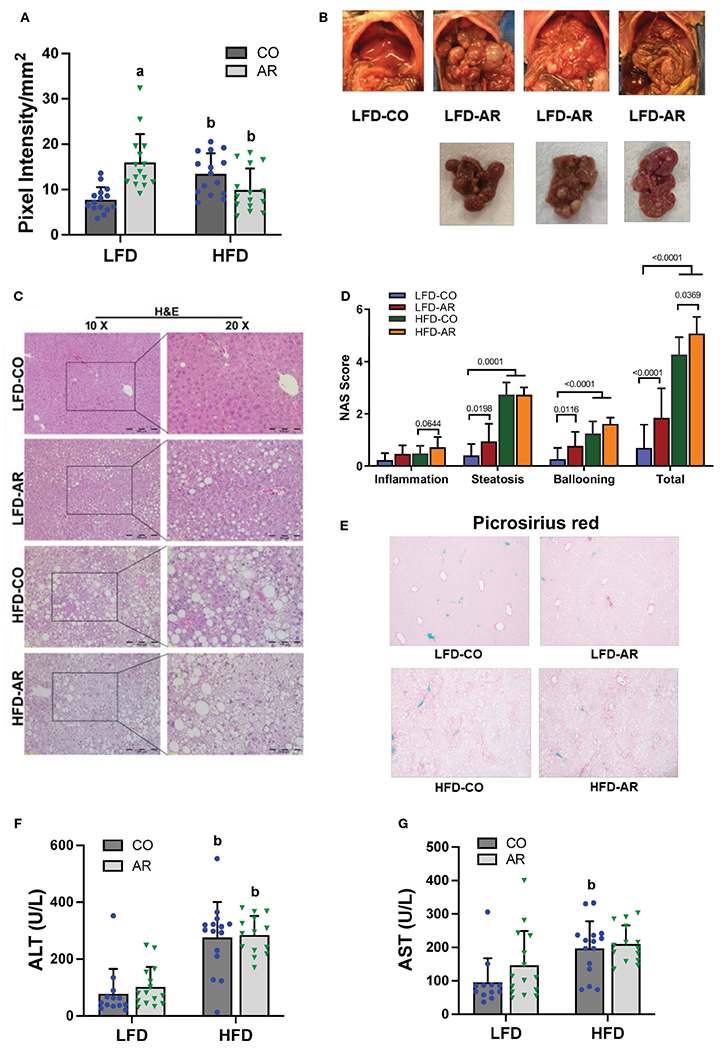
**(A)** Liver echogenicity was measured at week 23 by non-invasive ultrasound using the Vevo 3100 Imaging System and calculated using the Vevo LAB software. Data are expressed as pixel intensity per millimeter squared area. **(B)** Gross images of animal livers obtained at the end of the study period demonstrated visible tumor development in the LFD-AR group. No images were taken for isolated animal livers in the LFD-CO group during euthanasia and unavailable for comparison. **(C)** H&E staining on liver sections were performed to assess steatosis development and **(D)** NAFLD Activity Score (NAS) was analyzed in each H&E-stained slide. NAS was calculated by the sum of scores for steatosis (0–3), lobular inflammation (0–3), and hepatocyte ballooning (0–2). **(E)** Picrosirius red staining was performed on liver sections to assessed collagen deposition and fibrosis. (**F**, **G**) Plasma ALT and AST activity levels were measured using the Piccolo Xpress Chemistry Analyzer. Values are mean ± SD; *p* <0.05, a - Aroclor 1260 exposure effect, b - diet effect, c - interaction between exposure and diet.

Liver ultrasound was performed on week 23 of the study period to monitor for fatty liver development. Animal livers were evaluated by examining presence of roughened liver texture and increased echogenicity using this non-invasive method. While mice in the HFD groups manifested significantly higher liver echogenicity compared to the LFD-CO group (Figure 3A), Aroclor 1260 exposure also led to increased liver echogenicity in the LFD-fed mice. In concordance with the observed increased liver echogenicity for the LFD-AR group at 23 weeks, some liver tissues (n = 4) from the LFD-AR group acquired at the end of the study period (31 weeks) also displayed visible tumor burden (Figure 3B). No visible tumors were detected in the animal livers from LFD-CO, HFD-CO nor HFD-AR groups.

Histological staining of liver sections was performed to determine steatosis (H&E and Oil Red O staining) and fibrosis (Picrosirius red staining) development. When compared to the LFD-CO group, mild to moderate steatosis was observed in the other three experimental groups (Figure 3C and Supplemental Figure 2A). H&E sections were scored for degree of inflammation, steatosis and hepatocyte ballooning contributing to an overall NAFLD activity score (NAS) for the four groups (Figure 3D). Steatosis was significantly increased in HFD-fed mice while inflammation was significantly increased only in HFD-AR exposed mice. In contrast to our previous results with the short term (12-week) exposure, Aroclor 1260 exposure in the current study increased steatosis in LFD-fed mice. However, HFD plus Aroclor 1260 exposure showed no additional measures of steatosis above the contribution of HFD alone. LFD-AR mice also had more ballooned hepatocytes than LFD-CO mice, while a significant increase in ballooned hepatocytes was also evident in HFD-fed mice. NAS scores were significantly increased in response to both HFD feeding and Aroclor 1260 exposure (Figure 3D). Hepatic lipid assessment demonstrated that HFD feeding only led to increased cholesterol levels while hepatic triglyceride levels were increased in the HFD-AR group with an interaction effect between HFD feeding and Aroclor 1260 exposure (Supplemental Figures 2B, C). To explore the effects on fibrosis, Picrosirius red staining for collagen deposition was performed (Figure 3E). Increased collagen deposition visualized as fibrous septa was observed in the HFD groups irrespective of Aroclor 1260 exposure. Likewise, liver enzymes, namely, ALT and AST showed significant HFD-dependent increases in plasma levels but no differences attributable to Aroclor 1260 exposure (Figures 3F, G).

### Effects of Aroclor 1260 and HFD on hepatic pro-inflammatory and pro-fibrotic gene expression

Hepatic gene transcript expression levels for various markers of inflammation and injury were assessed and fold induction, relative to the LFD-CO group, was generated as depicted in Table 1. Transcript levels for genes encoding pro-inflammatory markers namely interleukin 18 (*Il-18*), macrophage inflammatory protein 1-alpha (*Mip1α*), and macrophage inflammatory protein 2-alpha (*Mip2α*) were elevated with HFD feeding. Aroclor 1260 exposure showed no changes on gene induction for any of the pro-inflammatory markers measured. However, there was an interaction effect for hepatic *Mip2α* expression which was elevated in LFD-AR mice compared to LFD-CO mice. Expression of genes encoding proteins involved in tissue injury and regeneration including transforming growth factor beta 1 (*Tgfb1*), tissue inhibitor of metalloproteinases 1 (*Timp1*), matrix metallopeptidase 12 (*Mmp12*), matrix metallopeptidase 13 (*Mmp13*), and plasminogen activator inhibitor-1 (PAI-1, *Serpine1*) were examined for diet and exposure-related effects. The abundance of *Timp1*, *Mmp12*, and *Mmp13* transcripts significantly increased with HFD feeding only while no changes were noted for *Tgfb1* or *Serpine1* mRNA levels. Furthermore, transcript levels of genes encoding pro-fibrotic markers namely smooth muscle alpha-2 actin (*Acta2*), collagen type I alpha 1 chain (*Col1α1*), and collagen type III, alpha-1 (*Col3α1*) were examined. Aroclor 1260 exposure resulted in significantly increased *Acta2* transcript abundance in LFD-AR mice compared to LFD-CO mice while HFD increased *Col1α1* expression. No statistical differences were observed in *Col3α1* transcripts between any of the groups.

### Plasma cytokine, adipokine, and lipid levels

With phenotypical indications of enhanced steatosis, inflammation, and underlying tissue alterations observed, circulating levels of adipo-cytokines and lipids were examined (Table 2). Plasma levels of pro-inflammatory tumor necrosis factor alpha (TNFα) remained unchanged with diet and exposure while there was a HFD effect for increased IL-6 plasma and PAI-1 levels. With regards to adipokines, HFD feeding resulted in increased plasma leptin levels while a diet effect was also noted for plasma resistin levels but with no significant changes between groups. HFD feeding resulted in upregulated plasma cholesterol and high-density lipoproteins (HDL) levels and downregulated plasma triglyceride levels. Aroclor 1260 had no effect on any of the circulatory markers measured.

### Glucose metabolism and insulin resistance

**FIGURE 4:**
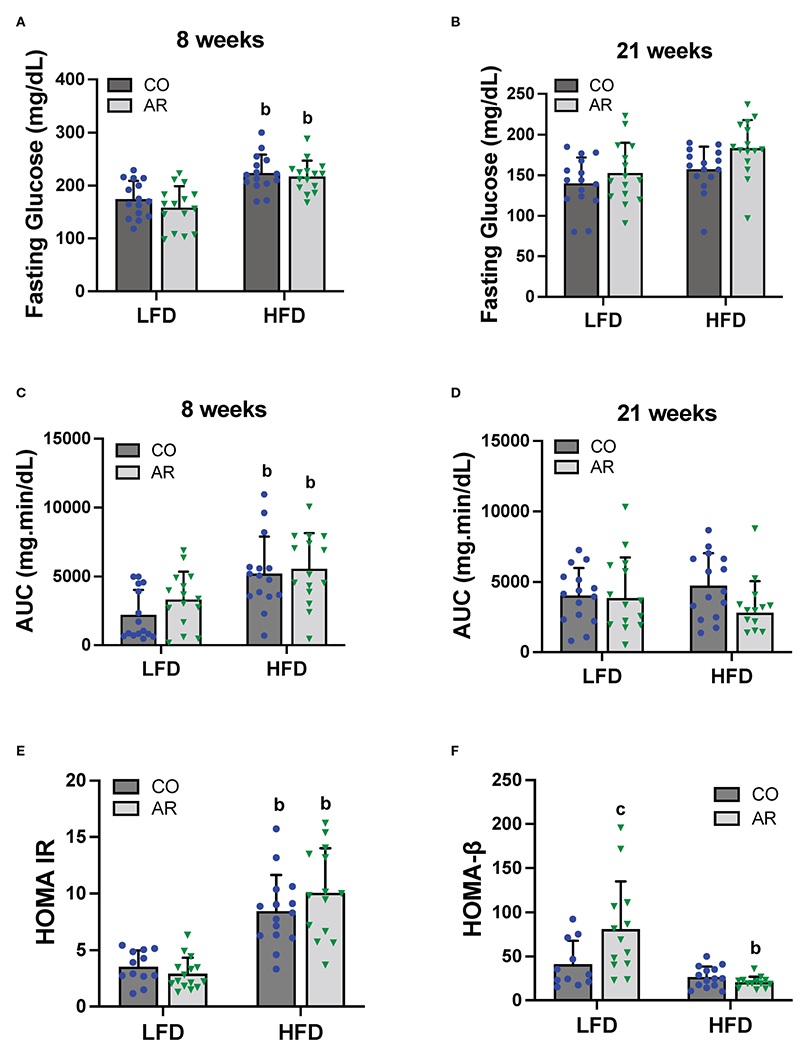
Glucose tolerance testing was performed at week 8 and week 21 of the study period. **(A, B)** Fasting blood glucose levels were measure at both time points. **(C, D)** Area under the curve (AUC) was calculated based on blood glucose levels measured (post glucose challenge) over a period of 2 hours for both time points. **(E, F)** Homeostasis model assessment for insulin resistance and β-cell function was calculated based on fasting blood glucose and insulin levels. Values are mean ± SD; *p* <0.05, a - Aroclor 1260 exposure effect, b - diet effect, c - interaction between exposure and diet.

Glucose tolerance testing was performed at week 8 and week 21 of the study period. Fasting blood glucose levels for HFD-fed mice were significantly elevated at 8 weeks post Aroclor 1260 exposure (Figure 4A). These elevated levels returned to baseline by 21 weeks post exposure although the HFD-AR group still showed a trend for increased glucose levels (Figure 4B). HFD feeding led to altered glucose uptake (increased AUC) at week 8 (Figure 4C and Supplemental Figure 3A) while no differences in AUC were observed at 21 weeks post exposure between groups (Figure 4D and Supplemental Figure 3B). Aroclor 1260 exposure had no impact on glucose uptake/clearance in both diet groups. Fasting blood glucose and insulin levels were measured at the end of the study period (Supplemental Figures 3C, D). Using these values, HOMA-IR was calculated to determine insulin sensitivity as related to diet and Aroclor 1260 exposure in this model (34). As previously observed (11), HFD-fed mice had significantly higher HOMA-IR levels (Figure 4E) compared to LFD-fed mice although Aroclor 1260 exposure did not impact HOMA-IR. Analysis of QUICKI which is another measure of insulin sensitivity were consistent with the HOMA-IR results (Supplemental Figure 3E). HOMA-β was calculated to determine pancreatic β-cell secretory function (34, 35). Surprisingly, Aroclor 1260 exposure increased HOMA-β in LFD-AR mice compared to the LFD-CO group whereas HFD-AR mice had the lowest HOMA-β scores, reflecting reduced β-cell function in the Aroclor 1260 group with HFD feeding (Figure 4F). An interaction effect between diet and Aroclor 1260 exposure was also observed for HOMA-β. The higher HOMA-IR values coupled with lowered HOMA-β values in HFD-fed mice suggest hepatic insulin resistance and pancreatic β-cell dysfunction with HFD feeding, while the improved HOMA-β scores with unchanged HOMA-IR values in the LFD-AR mice suggest a disconnect between insulin secretion and glucose metabolism as seen in our previous studies (11, 36).

### Assessment of hepatic genes involved in energy metabolism, liver injury, and oxidative stress

**FIGURE 5:**
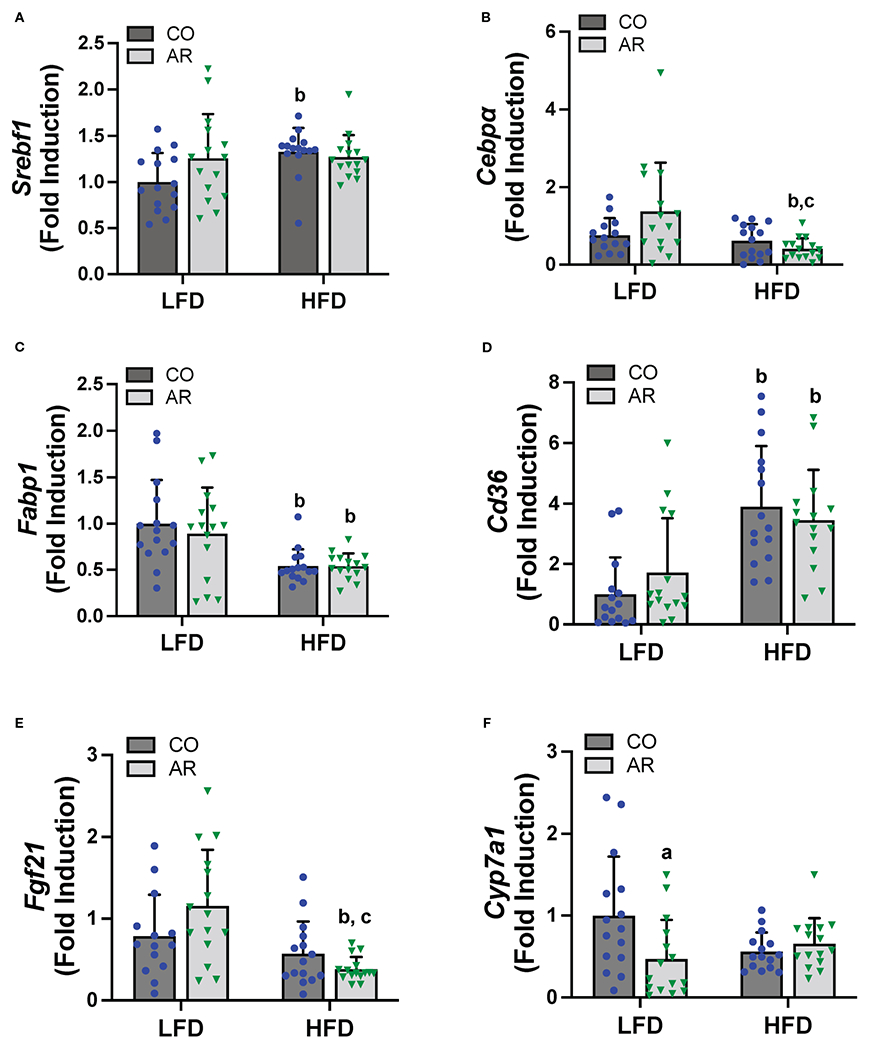
Hepatic mRNA expression for genes involved in lipid metabolism, namely **(A)** *Srebf1*, **(B)** *Cebpα*), **(C)** *Fabp1* and **(D)** *Cd36* were measured using RT-PCR. In addition, mRNA levels for genes encoding the hepatokine **(E)** *Fgf21* and enzyme involved in cholesterol/bile acid metabolism **(F)** *Cyp7a1* were measured. Values are mean ± SD; *p* <0.05, a - Aroclor 1260 exposure effect, b - diet effect, c - interaction between exposure and diet.

Hepatic expression of genes encoding proteins involved in hepatic energy metabolism, oxidative stress and liver injury were assessed. Firstly, mRNA levels of two transcription factors that regulate lipid metabolism including fatty acid synthesis, namely sterol regulatory element binding transcription factor (*Srebf1*) and CCAAT/enhancer-binding protein alpha (*Cebpα*) were assessed. HFD significantly increased *Srebf1* and decreased *Cebpα* expression while an interaction effect for decreased *Cebpα* expression in the HFD-AR group was also noted (Figures 5A, B). Expression levels of fatty acid binding protein 1 (*Fabp1*) and fatty acid translocase (*Cd36*) were also examined. HFD significantly decreased *Fabp1* and increased *Cd36* expression levels while exposure had no impact on the transcript abundance of either gene in either diet group (Figures 5C, D). HFD feeding also showed a trend for decreased mRNA levels for the enzyme involved in lipolysis, namely patatin like phospholipase domain containing 3 (*Pnpla3*) (Supplemental Figure 4A). In addition, HFD feeding decreased the hepatic expression of the hepatokine important for normal liver physiology, namely, fibroblast growth factor 21 (*Fgf21*) in the HFD-AR group (Figure 5E). Effects on bile acid metabolism were explored by examining cytochrome P450, family 7, subfamily a, member 1 (*Cyp7a1*), a protein coding gene for regulation of cholesterol conversion to bile acids. Notably, *Cyp7a1* mRNA levels were significantly reduced in LFD-AR mice compared to LFD-CO mice, implicating potential changes in cholesterol and bile acid metabolism (Figure 5F). Lastly, hepatic expression for genes encoding markers of oxidative stress and liver injury were examined. HFD feeding resulted in increased alpha fetoprotein (*Afp*) mRNA levels while other markers of oxidative stress including oxidoreductase heme oxygenase 1 (*Hmox1*), heat shock protein (*Hsp90b1*), and golgi membrane protein 1 (*Golm1*) were either changed with HFD feeding only or unchanged between groups (Supplemental Figures 4B–E).

### Hepatic receptor activation by Aroclor 1260

**FIGURE 6:**
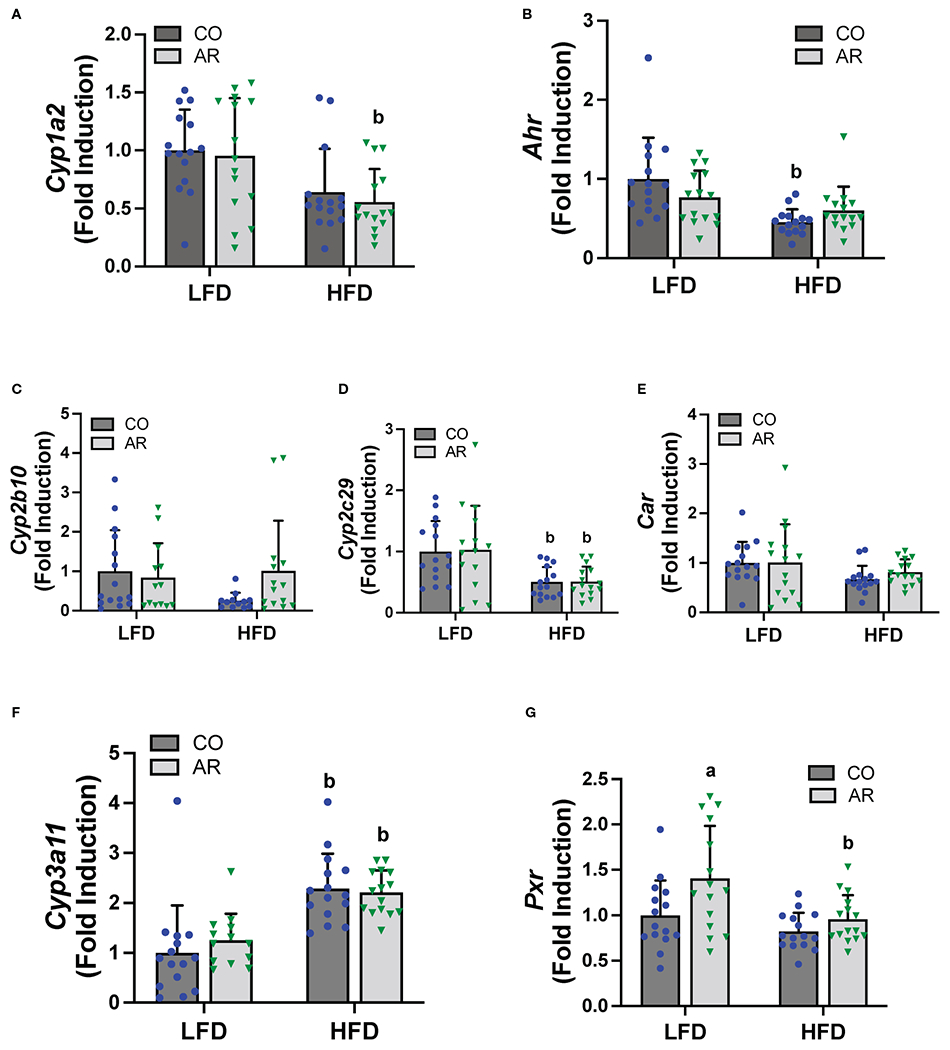
Hepatic mRNA expression for xenobiotic receptors and their target genes namely **(A, B)** *Cyp2b10* and *Ahr* **(C-E)** *Cyp2b10, Cyp2c29* and *Car*, and **(F, G)** *Cyp3a11* and *Pxr* were measured using RT-PCR. Values are mean ± SD; *p* <0.05, a - Aroclor 1260 exposure effect, b - diet effect, c - interaction between exposure and diet.

PCBs are known to activate hepatic xenobiotic receptors, including the AHR, CAR and the pregnane-xenobiotic receptor (PXR) and this is often assessed, in part, through induction of the cytochrome P450 receptor targets. We therefore examined the transcript abundance of the AHR, CAR and PXR as well as their target genes (Figure 6). Surprisingly, no cytochrome P450 induction was observed with Aroclor 1260 exposure for any of the receptor target genes which was counterintuitive to our previous findings. HFD feeding however led to decreased *Cyp1a2* (AHR target gene) in the HFD-AR group and decreased *Ahr* expression in the HFD-CO group (Figures 6A, B). Both *Car* and the prototypical CAR target gene, *Cyp2b10*, showed no change in expression attributable to either diet or Aroclor 1260 exposure while expression of the other CAR target gene, *Cyp2c29*, was decreased with HFD feeding (Figures 6C–E). In contrast, HFD feeding led to increased expression of *Cyp3a11* (PXR target gene) irrespective of exposure (Figure 6F). However, Aroclor1260 exposure led to increased *Pxr* gene expression in the LFD group (Figure 6G). Overall, these data implicated that the expected Aroclor 1260 receptor activation and subsequent gene induction may have disappeared after 31 weeks post exposure and suggest possible redistribution of PCB congeners to extra-hepatic tissue such as the adipose. A pilot assessment on adipose gene expression (Supplemental Figures 5A–F) demonstrated that while HFD feeding upregulated adipose *leptin* and *Tnfα* mRNA levels, Aroclor 1260 downregulated the HFD-induced expression of both genes. After accounting for body weight and white adipose tissue weight, *leptin* mRNA levels continued to be downregulated with Aroclor 1260 exposure particularly in the HFD group, implicating potential alterations in adipocyte gene expression that may need additional investigation.

### Effects of Aroclor 1260 and HFD feeding on echocardiogram

Echocardiography was performed at week 23 of the study period. These mid-study echocardiogram measurements indicated HFD-dependent alterations in cardiac function (Supplemental Figures 6A–F) including increased global longitudinal strain (GLS) and isovolumic relaxation time (IVRT). Alterations in GLS and IVRT can often precede chamber dimension differences and be predictive of cardiac dysfunction. However, no significant differences were observed with Aroclor 1260 exposure.

## Discussion

A plethora of studies ranging from epidemiologic reports to model systems have correlated exposures to PCBs, inclusive of both dioxin-like and non-dioxin-like congeners, with liver disease endpoints including elevated liver enzymes, steatosis, steatohepatitis, fibrosis, and liver cancer (11, 15, 16, 37). In addition to hepatic outcomes, PCB exposures have also been associated with cardiovascular outcomes including hypertension, endothelial dysfunction, and atherosclerosis (38–40). PCB disruption of the heart-liver axis has also been reported (21, 41). Established mechanisms for PCB-mediated TAFLD include hepatic xenobiotic receptor activation and subsequent endocrine, metabolic, and signaling disruption, in addition to other off-receptor target effects such as gut microbiota alterations and pancreatic toxicity (15). A considerable number of these toxicological assessments on PCB exposures and cardio-hepatic outcomes focused on shorter duration of exposures, or doses that were relatively higher compared to current environmental PCB exposure patterns. Thus, the current study was designed to further explore PCB toxicity outcomes pertinent to longer-term exposures with liver toxicity as the primary outcome, and effects on cardiovascular function as a secondary outcome. Aroclor 1260, in addition to a few dioxin-like congeners, was chosen because it most closely mimics non-occupational and bioaccumulated PCB exposure patterns, hence relevant to environmental PCB exposures today (42). The dose of PCBs used in the current study (20 mg/kg) reflected PCB doses used by our research group in previous studies (11, 12, 36, 43) which were designed to mimic the higher human PCB exposures seen in the Anniston Community Health Survey (ACHS) cohort, and PCB bioaccumulation patterns in humans seen with NHANES participants (14, 37, 44).

A major finding from the current study was the ability of Aroclor 1260 induce TAFLD/TASH outcomes, independent of high-fat feeding, when the duration of exposure was prolonged. Indeed, compared to our previous 12-week exposure studies where Aroclor 1260 exposure exacerbated steatohepatitis only in the conjunction with HFD feeding (11, 36), the current findings demonstrated that LFD-AR mice manifested increased levels of steatosis, NAS score and liver echogenicity. This suggests altered hepatocyte morphology in mice with longer duration of Aroclor 1260 exposure, without concurrent HFD administration. Significantly, some of the LFD-AR mice also exhibited liver tumors, suggestive of hepatocellular carcinoma development with longer-term Aroclor 1260 exposures. To our knowledge, this is the first report that Aroclor 1260, at environmentally relevant doses, induced hepatocellular carcinoma in a mouse model and strongly implicates how duration of exposures can lead to worsened TAFLD outcomes, even in the absence of a ‘second hit’ such as HFD feeding. However, these findings are not surprisingly given that PCBs are classified as International Agency for Research on Cancer (IARC) Group 1 carcinogens (45). Furthermore, recent epidemiologic studies have also reported associations between chronic PCB exposures and risk for liver cancer (46, 47).

Evaluation of potential mechanisms that may have played a role in PCB-mediated toxicity in the LFD-AR mice, including classic hepatic PCB receptor activation of CAR and PXR was not observed, although hepatic *Pxr* gene expression was increased. Moreover, neither increased insulin resistance nor significant pro-inflammatory cytokine induction (except for *Mip2α*) was observed. However, the LFD-AR mice exhibited increased hepatic transcript levels for the pro-fibrotic marker *Acta2*, suggesting a potential response to activated hepatic stellate cells which could occur during periods of liver tissue injury and recovery. Further, the transcript abundance for *Cyp7a1*, which catalyzes a rate-limiting step of bile acid synthesis, was reduced in our LFD-fed mice with Aroclor 1260 exposure. Recently, *Cyp7a1* has been explored as a potential target for NAFLD and HCC prognosis and therapeutic options (48, 49). Based on the reduction in *Cyp7a1* enzyme in our LFD-AR mice, we expected to see hypercholesterolemia; however, plasma cholesterol, triglycerides, and high-density lipoproteins were unaffected by Aroclor 1260 exposure particularly in the LFD-fed group. The impact of longer-term Aroclor 1260 exposure on FXR activity and bile acid metabolism, and how this may contribute to worsened TAFLD outcomes requires more in-depth analyses. Nonetheless, PCB exposures have been associated with human hepatocellular carcinoma development (46, 50). Intriguingly, pathway enrichment analysis and liquid liver biopsy generated from the differentially expressed circulating microRNAs associated with PCB exposures in the ACHS population demonstrated activation of hepatocellular carcinoma-associated pathways involving the tumor suppressor P53 (3). Overall, the mechanisms responsible for the observed PCB-associated hepatocellular carcinoma development in the current study, particularly focusing on carcinogenic pathways, are currently being investigated and findings will be disseminated in a separate publication.

Our previous 12-week study demonstrated increased circulating cholesterol and HDL levels with HFD feeding while triglyceride levels were unchanged (11). However, in the current study, HFD feeding also downregulated triglyceride levels which could be partially explained by enhanced lipid uptake to the liver (increased *Cd36* expression) in the HFD groups. Another notable finding in the current study is the lack of worsened steatohepatitis and fibrosis with longer-term Aroclor 1260 exposure with concurrent HFD feeding. While this observation contradicted our experimental hypothesis, it implicated the importance of considering redistribution of these lipophilic toxicants with time, especially in the context of high-fat intake and increased adiposity. In comparison to our previous 12-week exposure studies where hepatic proteomics findings indicated initiation of molecular mechanisms for liver fibrosis by Aroclor 1260 in HFD-fed mice (18), in the current study, no significant histological fibrosis was apparent with Aroclor 1260 exposure in HFD-fed mice. Although HFD feeding resulted in collagen deposition and fibrotic filaments in hepatic sections and increased transcript abundance of pro-fibrotic markers, Aroclor 1260 exposure did not exacerbated these endpoints. Previously, our group demonstrated that Aroclor 1260 indirectly activated hepatic stellate cells *in vitro via* hepatocyte-derived TGFβ and suggested this as a potential mechanism contributing to diet-induced steatohepatitis (18). In the current study, hepatocyte derived *Tgfb1* mRNA levels were not changed in response to either HFD or Aroclor 1260 exposure although a significant increase in *Timp1* and matrix metalloproteinases (*Mmp12* and *Mmp13*) expression was seen with HFD administration. Hepatic receptor activation was also absent in the Aroclor 1260-exposed mice, regardless of diet. While one explanation for this could be redistribution of PCBs to the adipose tissue or other compartments with time, another alternative explanation could be the single-dose administration of Aroclor 1260, so that the PCB congeners may have been metabolized with longer duration of exposure. Nonetheless, the majority of PCB congeners that constitute Aroclor 1260 are higher molecular weight congeners that are resistant to metabolism and bioaccumulate (12); however, there is no clear association between circulating levels of persistent organic pollutants and body fat mass (51), or how duration of exposure and time can influence this association. Therefore, analysis of tissue and circulating chemical levels are necessary to obtain a more comprehensive understanding in this area.

Other potential mechanisms currently postulated for both the worsened TAFLD outcomes with Aroclor 1260 exposure alone, and the absence of advanced fibrosis with HFD feeding, include evaluation of the C/EBP family of transcription factors and how PCBs modulate their expression or functionality in the context of steatohepatitis. Previously we reported that C/EBPα, C/EBPβ, and C/EBPδ were upregulated by Aroclor 1260 exposure but that PCB-HFD interactions downregulated C/EBPβ (18). In the current study, hepatic C/EBPα was downregulated with HFD feeding and an interaction effect between diet and exposure was observed. Importantly, the HFD-AR group also manifested increased hepatic triglyceride levels which may be associated with the downregulated hepatic *Cebpα* expression. Therapeutically, upregulated C/EBPα prevents tumor progression in advanced stages of human hepatocellular carcinoma and in various mouse tumor models (52–54), and deficient hepatic C/EBPα expression led to increased liver triglyceride accumulation in ob/ob mice (55). C/EBPα transcriptional regulation of genes involved in immune and inflammatory responses could be an indirect mechanism for PCB mediated liver toxicity when combined with overnutrition or hyper-caloric diets. Furthermore, limited information also exists for how overnutrition and PCB environmental exposures affect the epigenome (56–58) or epitranscriptome (59, 60). Our group recently identified 22 post-translational RNA modifications in mouse livers experimentally exposed to HFD and PCBs (PCB 126, Aroclor 1260, or PCB 126 + Aroclor 1260) for 12 weeks with 10 RNA modifications being statistically significant for PCB plus HFD-exposure when compared against diet alone (61). While these newer mechanisms of PCB toxicity are encouraging, further research is required in these areas to better understand PCB exposures and fibrosis development in conjunction with overnutrition.

The current findings provided novel insight into how PCB-mediated hepatotoxicity unfolds with time which is a major strength of the study; however, the study is not without limitations. Firstly, the study only examined C57BL/6J male mice and did not consider how biological sex could impact these findings. Our previous studies on PCBs and sex differences have suggested that females may be more susceptible to PCB-mediated hepatotoxicity, and that Aroclor 1260 exposure promoted antiestrogenic effects in both sexes (62). Additionally, Mayes et al. demonstrated more severe liver toxicity in females as compared to males in their studies examining chronic toxicity and carcinogenicity of four different polychlorinated biphenyl mixtures including Aroclor 1260 (63). To address this limitation, examination of prolonged PCB exposures in male and female models will be performed as a future direction. Another major limitation in the study is the introduction of experimental stressors including use of anesthesia, echocardiogram assessment and DEXA scan measurements during the study period (64–66). In addition to inducing changes in normal physiological responses such as body weight gain (Supplemental Figure 7), these experiments also required changes in animal housing facility during the mid-study period that could have potentially impacted gut microbiota composition and energy metabolism for these experimental animals. Furthermore, the effects of aging were not considered when analyzing the current longer-term toxicity indices, and age-adjustment will be performed in our future longer-term exposure studies. In addition, PCB measurements in the liver, plasma, and adipose tissue were not measured to assess PCB redistribution with time and with increased adiposity. These measurements are being performed and will shed further light on this topic. Lastly, a thorough assessment of cardiovascular parameters was not carried out in the current study. While the initial echocardiogram results suggest subtle or no PCB effects on the measured cardiovascular outcomes other than HFD effects, further investigation of longer-term PCB effects on cardio-metabolic endpoints including cardiac gene expression, vascular endothelial function, and physiology are warranted.

In summary, our study characterized the hepatic phenotype and accompanying metabolic disruptions including altered *de novo* lipogenesis, lipid transport and storage, and promotion of fibrotic markers in mice concurrently exposed to dietary insult (HFD) and an environmentally relevant PCB mixture (Aroclor 1260). Hepatic tumors visualized in our LFD-AR mice were an unexpected finding and carcinogenic mechanisms are currently being investigated. A clearer picture of PCB metabolism, congener specific bioaccumulation patterns, and site-specific toxicity is necessary to fully understand metabolic disruptions seen with PCB exposure and how the mitigating factors of biological sex, hyper-caloric diets, length of exposure or conformational structure of PCBs work collectively to progress TAFLD to TASH and lead to chronic liver injury outcomes such as fibrosis, cirrhosis, or cancer. Continued research focusing on epigenetic and epitranscriptomic regulation of the hepatic proteome and crosstalk between the liver, gut, adipose, and multiple organ systems can further our knowledge on how endocrine and metabolism disrupting chemicals that are still present in our environment may continue to affect underlying health and disease pathologies. Importantly, how do these chemicals along with lifestyle factors such as hyper-caloric intake, alcohol consumption, smoking, etc. work together to compromise human health? Future studies in our laboratory will explore these complicated lifestyle factors and environmental exposure interactions.

## Supplementary Material

Supplementary Material

## Figures and Tables

**TABLE 1:** RT-PCR was performed to assess hepatic expression for genes involved in inflammation and fibrosis.

Gene (Fold Induction)	LFD-CO	LFD-AR	HFD-CO	HFD-AR
*Il-6*	1.00 ± 2.68	0.65 ± 0.70	0.22 ± 0.10	0.15 ± 0.05
*Tnfα*	1.00 ± 2.30	1.45± 2.08	1.11 ± 0.62	1.08 ± 0.34
*Il-18*	1.00 ± 0.80	1.96 ± 2.36	2.29 ± 1.83^b^	2.69 ± 1.73
*Mip1α*	1.00 ± 1.20	1.62 ± 1.87	2.71 ± 1.95^b^	2.84 ± 0.92
*Mip2α*	1.00 ± 0.94	1.84 ± 2.33^c^	3.56 ± 1.65^b^	2.69 ± 0.95
*Tgfb1*	1.00± 0.96	1.10 ±0.80	1.35 ± 0.57	1.45 ±0.36
*Timp1*	1.00± 3.38	1.70 ± 3.97	7 ± 5.52^b^	7.15 ±3.15^b^
*Mmp12*	1.00 ± 2.77	4.57 ±8.88	24.9± 11.5^b^	27.1± 10.3^b^
*Mmp13*	1.00 ± 1.67	6.59 ± 10.3	15.8 ± 10.1^b^	19.1 ± 9.35^b^
*Serpine1*	1.00 ± 1.25	1.44 ± 2.34	1.28 ± 1.12	1.66 ± 1.26
*Acta2*	1.00 ±0.63	2.85 ± 2.88^a^	2.35 ± 1.25	2.59± 1.33
*Col1α1*	1.00± 2.46	1.53 ± 2.20	3.52 ± 1.70^b^	4.23 ± 1.61^b^
*Col3α1*	1.00 ± 3.82	0.455 ± 1.07	2.32 ± 1.55	2.57 ± 1.65^b^

Values are mean ± SD; p <0.05,

a - Aroclor 1260 exposure effect,

b - diet effect,

c - interaction between exposure and diet.

**TABLE 2:** Plasma adipo-cytokines and lipid levels were measured using either the Luminex System or Piccolo Chemistry Analyzer.

Analyte	LFD-CO	LFD-AR	HFD-CO	HFD-AR
IL-6 (pg/mL)	28.5 ± 34.0	31.6 ± 27.1	10.7 ± 5.7	15.9 ± 12.9
TNFα (pg/mL)	7.3 ± 8.7	62.6 ± 148	7.3 ± 7.4	6.8 ± 7.0
PAI-1 (pg/mL)	1730.4± 1392.4	2456.5 ± 2015.4	4113.3 ± 3297.3	4265.3 ± 3476.1
Leptin (pg/mL)	5030.6± 6726.9	7716.4 ± 6125.6	15452.1 ± 6719.6^b^	12979.5 ± 4521.8
Resistin (pg/mL)	1412.7± 780.0	1586.7 ± 422.9	1843.1 ± 649.6	1902.7± 699.1
Cholesterol (mg/dL)	74.1 ± 5.6	75.3 ± 7.1	145.1 ±14.1^b^	170.0 ± 12.6^b^
Triglycerides (mg/dL)	48.6 ± 3.3	48.1 ± 4.6	33.2 ± 2.3^b^	32.2 ± 1.8^b^
HDL (mg/dL)	57.1 ± 4.0	55.7± 6.5	>100^b^	>100^b^

Values are mean ± SD; p <0.05,

a - Aroclor 1260 exposure effect,

b - diet effect,

c - interaction between exposure and diet.

## Data Availability

The original contributions presented in the study are included in the article/Supplementary Material. Further inquiries can be directed to the corresponding authors.

## Highlights

- Aroclor 1260 (31 week-exposure) caused steatohepatitis and liver tumors in mice fed a low-fat diet.
- Aroclor 1260 (31 week-exposure) did not exacerbate NAFLD nor cause liver tumors in high fat diet-fed mice.
- Hepatic xenobiotic receptor activation by Aroclor 1260 was absent at 31 weeks post exposure.
