# Supplementary material for: Investigating the effects of long-term Aroclor 1260 exposure on fatty liver disease in a diet-induced obesity mouse model
Source: Front Gastroenterol (Lausanne). 2023 May 12;2:1180712. doi: 10.3389/fgstr.2023.1180712 (PMC10327714; doi:10.3389/fgstr.2023.1180712)
Supplement: Supplementary file 1 [file DataSheet_1.pdf]

Investigating the Effects of Long-Term Aroclor 1260 Exposure on Fatty Liver Disease in a Diet-Induced Obesity Mouse Model

Kimberly Z. Head, Oluwanifemi E. Bolatimi, Tyler C. Gripshover, Min Tan, Yan Li, Timothy N. Audam, Steven P. Jones, Carolyn M. Klinge, Matthew C. Cave, and Banrida Wahlang

Supplemental Table 1

Diet Composition.

	Low Fat Diet (LFD) TD.06416		High Fat Diet (HFD) TD.88137	
**Component**	**%kCal**	**Source**	**%kCal**	**Source**
**Protein**	20	Casein	15.2	Casein
**Carbohydrate**	69.8	Sucrose/Corn starch	42.7	Sucrose/Corn starch
**Fat**	10.2	Anhydrous Milkfat	42	Anhydrous Milkfat
**kCal/g**	3.7		4.5	

Low fat diet (LFD). Casein 210.0 g/kg, L-Cystine 4.0 g/kg, Sucrose 325.0 g/kg, Corn Starch 280.0 g/kg, Maltodextrin 50.0 g/kg, Lard 20.0 g/kg, Soybean Oil 20.0 g/kg, Cellulose 37.15 g/kg, Mineral Mix, AIN-93G-MX (94046) 35.0 g/kg, Calcium Phosphate, dibasic 2.0 g/kg, Vitamin Mix, AIN-93-VX (94047) 15.0 g/kg, Choline Bitartrate 2.75 g/kg.

High fat diet (HFD). Casein 195.0 g/kg, DL-Methionine 3.0 g/kg, Sucrose 341.46 g/kg, Corn Starch 150 g/kg, Maltodextrin 100.0 g/kg, Anhydrous Milkfat 210.0 g/kg, Cholesterol 1.5 g/kg, Cellulose 50 g/kg, Mineral Mix, AIN-76 (170915) 35.0 g/kg, Calcium Carbonate 4.0 g/kg, Vitamin Mix, Teklad (40060) 10.0 g/kg, Ethoxyquin, antioxidant 0.04 g/kg.

Supplemental Table 2

Product information for Taqman primers used for RT-PCR.

Gene/Primer name	Product information
Alpha 2 actin (*Acta2*)	Mm01546133_m1
Adiponectin (*Adipoq*)	Mm00456425_m1
Alpha Fetoprotein (*Afp*)	Mm00431715_m1
Aryl hydrocarbon receptor (*AhR*)	Mm00478932_m1
Constitutive androstane receptor (*Car*)	Mm01283978_m1
CCAAT/enhancer-binding protein alpha (*Cebpα*)	Mm00514283_m1
Cluster of Differentiation 36 (*Cd36*)	Mm01135198-m1
Collagen type I, alpha 1 (*Colα1*)	Mm00801666_g1
Collagen type III, alpha 1 (*Col3α1*)	Mm00802300_m1
Cytochrome P450 family 1, subfamily a, polypeptide 2 (*Cyp1a2*)	Mm00487224_m1
Cytochrome P450 family 2, subfamily b, polypeptide 10 (*Cyp2b10*)	Mm01972453-s1
Cytochrome P450 family 2, subfamily c, polypeptide 29 (*Cyp2c29*)	Mm00725580_m1
Cytochrome P450 family 3, subfamily a, polypeptide 11 (*Cyp3a11*)	Mm007731567-m1
Cytochrome P450 family 7, subfamily a, polypeptide 1 (*Cyp7a1*)	Mm00484150_m1
DNA-damage inducible transcript 3 (*Ddit3*)	Mm00492097_m1
Fatty Acid Binding Protein (*Fabp1*)	Mm00443440-m1
Fibroblast growth factor 21 (*Fgf21*)	Mm00840165_g1
Glyceraldehyde-3-Phosphate Dehydrogenase (*Gapdh*)	4351309
Golgi membrane protein 1 (*Golm1*)	Mm00550918_m1

Heat shock protein 90, beta (Grp94), member 1 (*Hsp90b1*)	Mm00441926_m1
Heme oxygenase 1 (*Hmox1*)	Mm00516005_m1
Interleukin 6 (*Il-6*)	Mm00446190-m1
Interleukin 18 (*Il-18*)	Mm00434226_m1
Leptin (*Lep*)	Mm00434759-m1
Macrophage inflammatory protein 1-alpha (*Mip1α*)	Mm00441259_g1
Macrophage inflammatory protein 2-alpha (*Mip2α*)	Mm00436450_m1
Matrix metallopeptidase 12 (*Mmp12*)	Mm00500554_m1
Matrix metallopeptidase 13 (*Mmp13*)	Mm00439491_m1
Patatin-like phospholipase domain containing protein-3 (Pnpla3)	Mm00504420_m1
Pregnane xenobiotic receptor (*Pxr*)	Mm01344139_m1
Serpine1	Mm00435858_m1
Sterol regulatory element binding transcription factor (*Srebf1*)	Mm00550338_m1
Tissue inhibitor of metalloproteinase 1 (*Timp1*)	Mm01341361_m1
Transforming growth factor, beta 1 (*Tgfb1*)	Mm01178820_m1
Tumor necrosis factor alpha (*Tnfα*)	Mm00443258_m1

Supplemental Table 3

A list of *p*-values generated from two-way ANOVA analysis and Tukey's *post-hoc* test is provided below for body weight (BW) and body composition measurements. Significance was set at 0.05. LW - liver weight, WAT - white adipose tissue.

Endpoint	Interaction	Diet	Exposure	LFD-CO vs. LFD-AR	LFD-CO vs. HFD-CO	LFD-AR vs. HFD-AR	HFD-CO vs. HFD-AR
**% Inc BW**	0.4786	**<0.0001**	0.2101	0.9788	**<0.0001**	**<0.0001**	0.5116
**LW:BW**	0.4725	**<0.0001**	0.2696	0.5493	**<0.0001**	**<0.0001**	0.9929
**Total Fat**	0.0837	**<0.0001**	0.2671	0.9638	**<0.0001**	**<0.0001**	0.686
**% Fat/BW**	0.6931	**<0.0001**	0.0894	0.7826	**<0.0001**	**<0.0001**	0.4428
**Lean Tissue**	0.7639	**0.0391**	**0.0178**	0.4365	0.3296	0.5790	0.2233
**WAT/BW (%)**	0.9177	**<0.0001**	0.1993	0.8325	**0.0041**	**0.0064**	0.7544

Supplemental Table 4

A list of *p*-values generated from two-way ANOVA analysis and Tukey's *post-hoc* test is provided below for liver endpoint measurements. Significance was set at 0.05. ALT - alanine aminotransferase, AST - aspartate aminotransferase.

Endpoint	Interaction	Diet	Exposure	LFD-CO vs. LFD-AR	LFD-CO vs. HFD-CO	LFD-AR vs. HFD-AR	HFD-CO vs. HFD-AR
**Liver Echogenicity**	**<0.0001**	0.8748	0.0636	**<0.0001**	**0.0092**	**0.0048**	0.1777
**Plasma ALT**	0.758	**<0.0001**	0.5065	0.9023	**<0.0001**	**<0.0001**	0.994
**Plasma AST**	0.3847	**0.0004**	0.1434	0.3672	**0.0104**	0.1584	0.9713
**Hepatic Triglycerides**	**0.0017**	**<0.0001**	0.3452	0.3539	**0.0001**	**<0.0001**	**0.0200**
**Hepatic Cholesterol**	0.3630	**<0.0001**	0.8984	0.9440	**0.0003**	**<0.0001**	0.8808

Supplemental Table 5

A list of *p*-values generated from two-way ANOVA analysis and Tukey's *post-hoc* test is provided below for glucose and insulin measurements. Significance was set at 0.05. AUC - area under the curve, HOMA-IR - homeostatic model assessment for insulin resistance, HOMA-β - homeostasis model assessment of beta cell function, QUICKI - quantitative insulin sensitivity check index.

Endpoint	Interaction	Diet	Exposure	LFD-CO vs. LFD-AR	LFD-CO vs. HFD-CO	LFD-AR vs. HFD-AR	HFD-CO vs. HFD-AR
**Fasting glucose-8 weeks**	0.5947	**<0.0001**	0.2206	0.5954	**0.0018**	**0.0002**	0.9593
**Fasting glucose-21 weeks**	0.4408	**0.0068**	**0.0273**	0.7187	0.4803	0.0649	0.1497
**AUC- 8 weeks**	0.5163	**<0.0001**	0.2214	0.5441	**0.0039**	**0.0489**	0.9761
**AUC- 21 weeks**	0.1562	0.7798	0.0898	0.9969	0.8459	0.6201	0.1258
**HOMA-IR**	0.1399	**<0.0001**	0.5161	0.936	**0.0002**	**<0.0001**	0.4131
**HOMA-β**	**0.011**	**<0.0001**	0.0544	**0.0138**	0.6391	**<0.0001**	0.9605
**Plasma Glucose**	**0.0450**	**<0.0001**	0.7572	0.3573	**0.0280**	**<0.0001**	0.6020
**Plasma Insulin**	0.6836	**<0.0001**	0.5146	0.8748	**0.0046**	**0.0008**	0.9981
**QUICKI**	0.0829	**<0.0001**	0.4411	0.2936	**0.0009**	**<0.0001**	0.8935

Supplemental Table 6

A list of *p*-values generated from two-way ANOVA analysis and Tukey's *post-hoc* test is provided below for gene expression assays determined by RT-PCR. Significance was set at 0.05.

Gene	Interaction	Diet	Exposure	LFD-CO vs. LFD-AR	LFD-CO vs. HFD-CO	LFD-AR vs. HFD-AR	HFD-CO vs. HFD-AR
**Il-6**	0.694	0.079	0.567	0.902	0.418	0.759	0.999
**Tnfα**	0.560	0.756	0.615	0.867	0.997	0.920	0.999
**Il-18**	0.545	**0.031**	0.142	0.454	0.202	0.670	0.924
**Mip1α**	0.543	**0.001**	0.355	0.696	0.020	0.149	0.996
**Mip2α**	**0.040**	**0.000**	0.977	0.464	0.000	0.458	0.440
**Tgfb1**	0.993	0.063	0.572	0.979	0.545	0.537	0.977
**Timp1**	0.544	**0.000**	0.945	0.965	**0.000**	**0.003**	0.982
**Mmp12**	0.761	**0.000**	0.2240	0.700	**0.000**	**0.000**	0.914
**Mmp13**	0.609	**0.000**	0.051	0.297	**0.000**	**0.001**	0.723
**Serpine1**	0.941	0.544	0.313	0.867	0.962	0.981	0.909
**Acta2**	0.088	0.250	**0.028**	**0.037**	0.196	0.976	0.982
**Col1α1**	0.869	**0.000**	0.249	0.896	**0.006**	**0.004**	0.778
**Col3α1**	0.505	**0.005**	0.802	0.914	0.395	0.066	0.991
**Srebf1**	0.0755	0.0543	0.2611	0.1712	**0.0473**	0.9995	0.9637
**Cebpα**	**0.0326**	**0.0049**	0.2798	0.1101	0.9536	**0.0031**	0.861
**Fabp1**	0.5789	**<0.0001**	0.5602	0.8499	**0.0053**	**0.0456**	>0.9999
**Cd36**	0.1894	**<0.0001**	0.7593	0.656	**0.0001**	**0.0337**	0.888
**Fgf21**	**0.0273**	**0.0002**	0.4628	0.1634	0.6234	**0.0002**	0.6969

**Cyp7a1**	**0.013**	0.3032	0.0821	**0.017**	0.0631	0.7029	0.9426
**Hsp90b1**	0.9019	**<0.0001**	0.1466	0.7762	**<0.0001**	**<0.0001**	0.6737
**Afp**	0.3274	**<0.0001**	0.4572	0.9983	**0.0002**	**<0.0001**	0.6119
**Cyp1a2**	0.8569	**0.0004**	0.5123	0.9865	0.0616	**0.0329**	0.9333
**Ahr**	0.0426	**0.0003**	0.6293	0.2794	**0.0005**	0.5833	0.6765
**Cyp2b10**	0.0863	0.273	0.2591	0.9715	0.1961	0.9684	0.1992
**Car**	0.5888	**0.0388**	0.5355	**>0.9999**	0.2478	0.6836	0.8422
**Cyp2c29**	0.9327	**<0.0001**	0.888	0.9985	**0.0257**	**0.0187**	>0.9999
**Cyp3a11**	0.3657	**<0.0001**	0.6338	0.7691	**<0.0001**	**0.0032**	0.9893
**Pxr**	0.1815	**0.0027**	**0.009**	**0.0286**	0.5924	**0.0127**	0.7734
**Golm1**	0.1843	**<0.0001**	0.9808	0.8091	**<0.0001**	**<0.0001**	0.7385
**Hmox1**	0.9864	**0.0109**	0.6210	0.9834	0.2502	0.2608	0.9864
**Pnpla3**	0.2840	**0.0120**	0.2367	0.3810	0.0558	0.7078	0.9998
**Adipose Tnfa**	**0.0431**	**<0.0001**	**0.0023**	0.8588	**0.0003**	0.3969	**0.0021**
**Adipose Adipoq**	0.2240	0.1339	0.7852	0.9099	0.9970	0.2077	0.7011
**Adipose Leptin**	**0.0163**	**<0.0001**	**0.0010**	0.8977	**<0.0001**	0.0605	**0.0004**
**Adipose Chop/Ddit3**	0.3405	0.0591	0.0383	0.8497	0.1852	0.9022	0.1300

Supplemental Table 7

A list of *p*-values generated from two-way ANOVA analysis and Tukey's *post-hoc* test is provided below for plasma analyte measurements. Significance was set at 0.05. PAI-1 - plasminogen activator inhibitor type 1, HDL - high density lipoprotein.

**Analyte**	**Interact**	**Diet**	**Exposure**	**LFD-CO vs. LFD-AR**	**LFD-CO vs. HFD-CO**	**LFD-AR vs. HFD-AR**	**HFD-CO vs. HFD-AR**
**IL-6**	0.859	**0.006**	0.485	0.982	0.156	0.440	0.070
**TNFα**	0.151	0.152	0.159	0.187	0.999	0.999	0.187
**PAI-1 total**	0.681	**0.004**	0.530	0.881	0.084	0.0589	0.340
**Leptin**	0.107	**0.000**	0.946	0.6246	**0.000**	**0.004**	**0.005**
**Resistin**	0.753	**0.031**	0.490	0.884	0.280	0.179	0.704
**Cholesterol**	0.272	**0.000**	0.227	0.9998	**0.000**	**0.000**	**0.000**
**Triglycerides**	0.938	**0.000**	0.822	0.9996	**0.008**	**0.005**	**0.008**
**HDL**	0.463	**0.000**	0.677	0.996	**0.000**	**0.000**	**0.000**

Supplemental Table 8

A list of *p*-values generated from two-way ANOVA analysis and Tukey's *post-hoc* test is provided below for echocardiogram endpoint measurements. Significance was set at 0.05. GLS - Global longitudinal strain, IVRT - Isovolumic relaxation time, ESV - Left ventricular end systolic volume.

Endpoint	Interact	Diet	Exposure	LFD-CO vs. LFD-AR	LFD-CO vs. HFD-CO	LFD-AR vs. HFD-AR	HFD-CO vs. HFD-AR
**Heart Rate**	0.8929	**0.0030**	0.3445	0.9381	0.1671	0.1133	0.8677
**Cardiac Output**	0.6780	**0.0171**	0.8898	0.9791	0.4784	0.1882	0.9973
**GLS**	0.6343	**0.0014**	0.5404	0.8659	0.1878	0.0429	0.9997
**IVRT**	0.0731	**0.0034**	0.0618	0.0510	0.8209	**0.0057**	>0.9999
**Ejectile Fraction**	0.7872	**0.0126**	0.4793	0.8984	0.1950	0.3701	0.9894
**ESV**	0.8209	**0.0453**	0.4233	0.8842	0.3822	0.5750	0.9766

Supplemental Figure 1

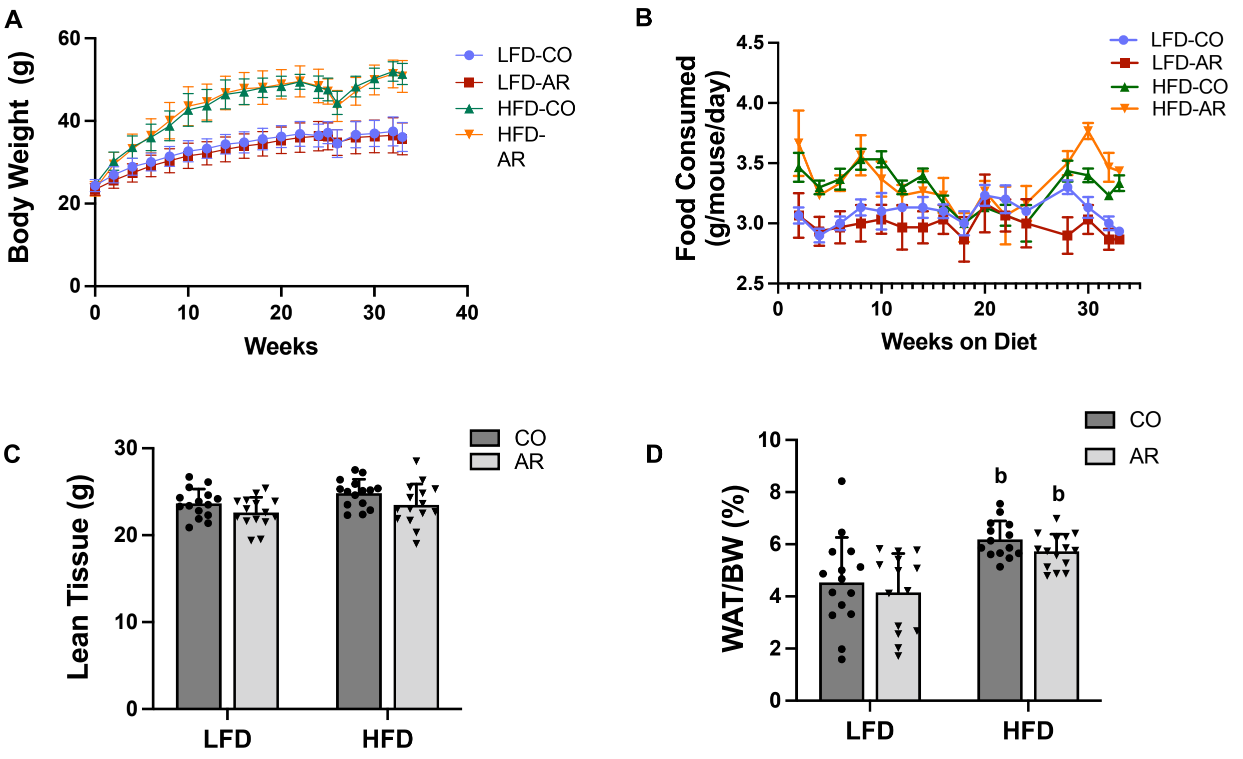

Supplemental Figure 1. Aroclor 1260 effects on body weight, food consumption and body composition. (A) Body weight was measured biweekly and (B) Food consumption was calculated throughout the 31-week study. (C) Lean tissue weight was measured using DEXA scanning for all mice. (D) Epididymal adipose weight, representative of white adipose tissue (WAT) as a percentage of body weight was determine at the end of the study period. Values are mean ± SD; *p* <0.05, a - Aroclor 1260 effect, b - diet effect, c - interaction effect.

Supplemental Figure 2

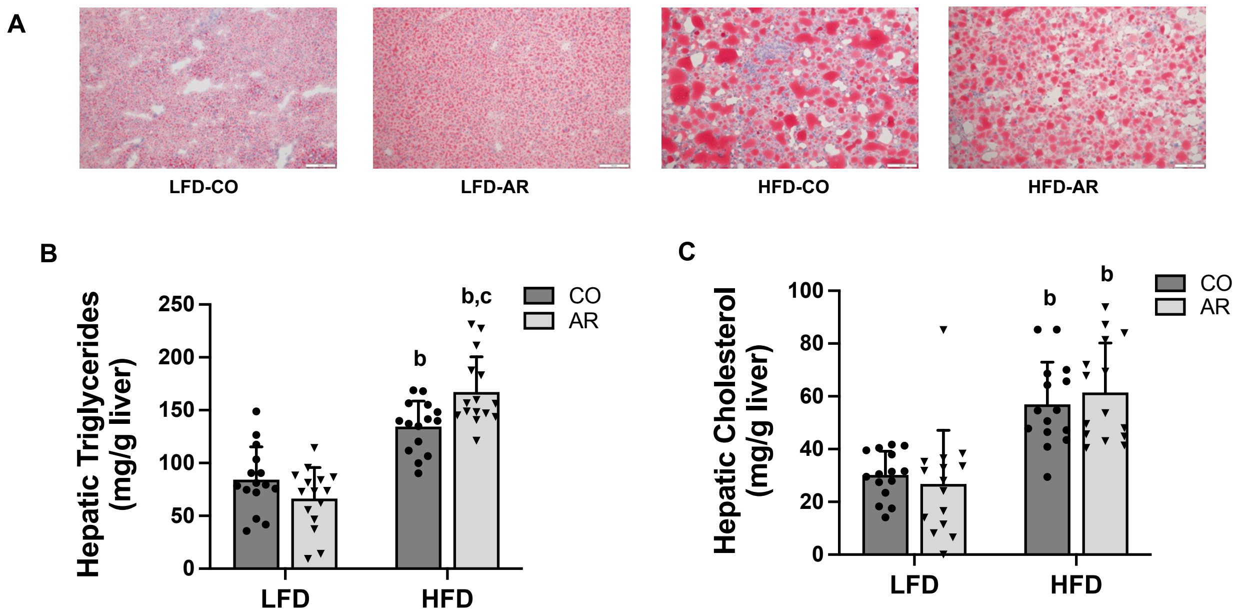

Supplemental Figure 2. Aroclor 1260 effects on hepatic steatosis. (A) Representative images of Oil Red O staining on frozen liver sections demonstrated mild lipid droplets in the LFD-AR group and pronounced lipid accumulation in the HFD groups. Hepatic levels of (B) triglycerides and (C) cholesterol were quantified using colorimetric assays in all diet and exposure groups. Values are mean ± SD; p <0.05, a - Aroclor 1260 effect, b - diet effect, c - interaction effect.

Supplemental Figure 3

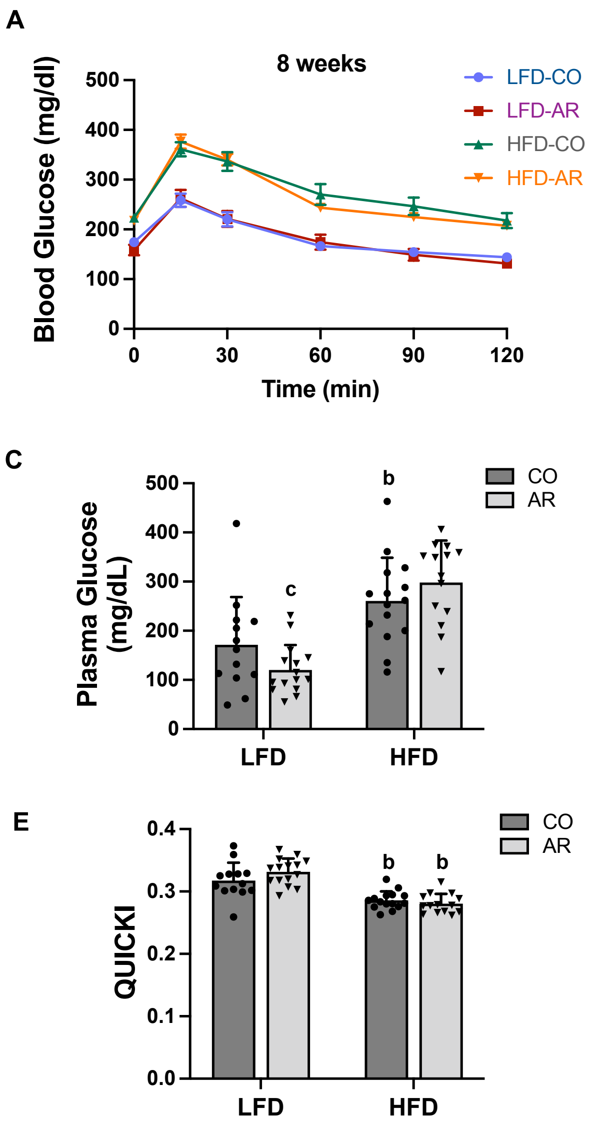

Supplemental Figure 3. Plasma glucose and insulin levels. Blood glucose levels were measured at (A) week 8 and (B) week 21 of study duration. (C) Measurements of plasma glucose at the end of the study period was conducted using the Piccolo Xpress Chemistry Analyzer. (D) Plasma insulin levels were measured using ELISA. (E) QUICKI assessment of insulin resistance was also calculated. Values are mean ± SD; p <0.05, a - Aroclor 1260 effect, b - diet effect, c - interaction effect.

<u>**Supplemental Figure 4**</u>

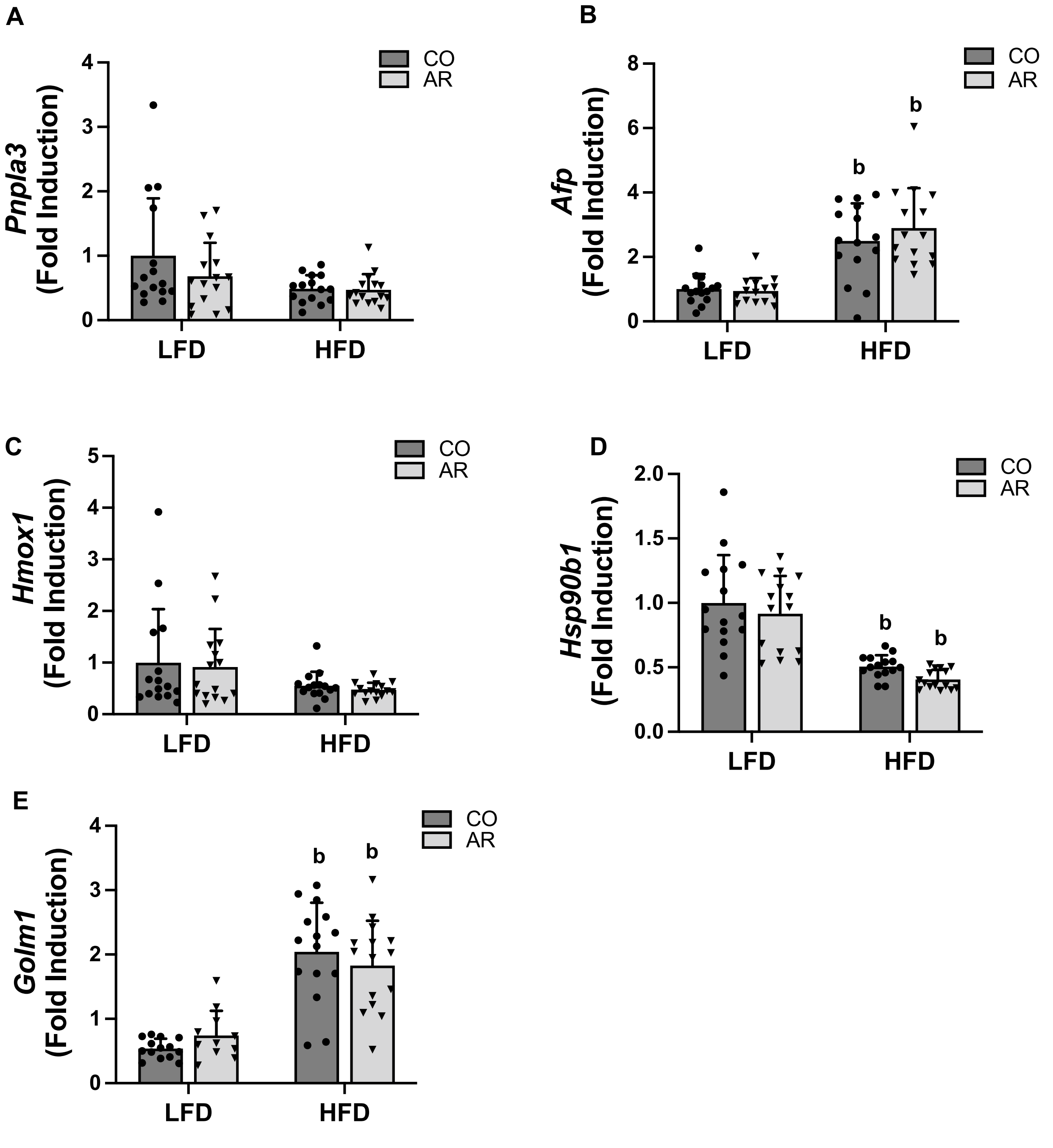

Supplemental Figure 4. Gene markers of lipolysis, oxidative stress, and liver injury. Hepatic mRNA levels for (A) *Pnpla3* (B) *Afp* (C) *Hmox1*, (D) *Hsp90b1*, and (E) *Golm1* were measured by RT-PCR. Values are mean ± SD; p <0.05, a - Aroclor 1260 effect, b - diet effect, c - interaction effect.

Supplemental Figure 5

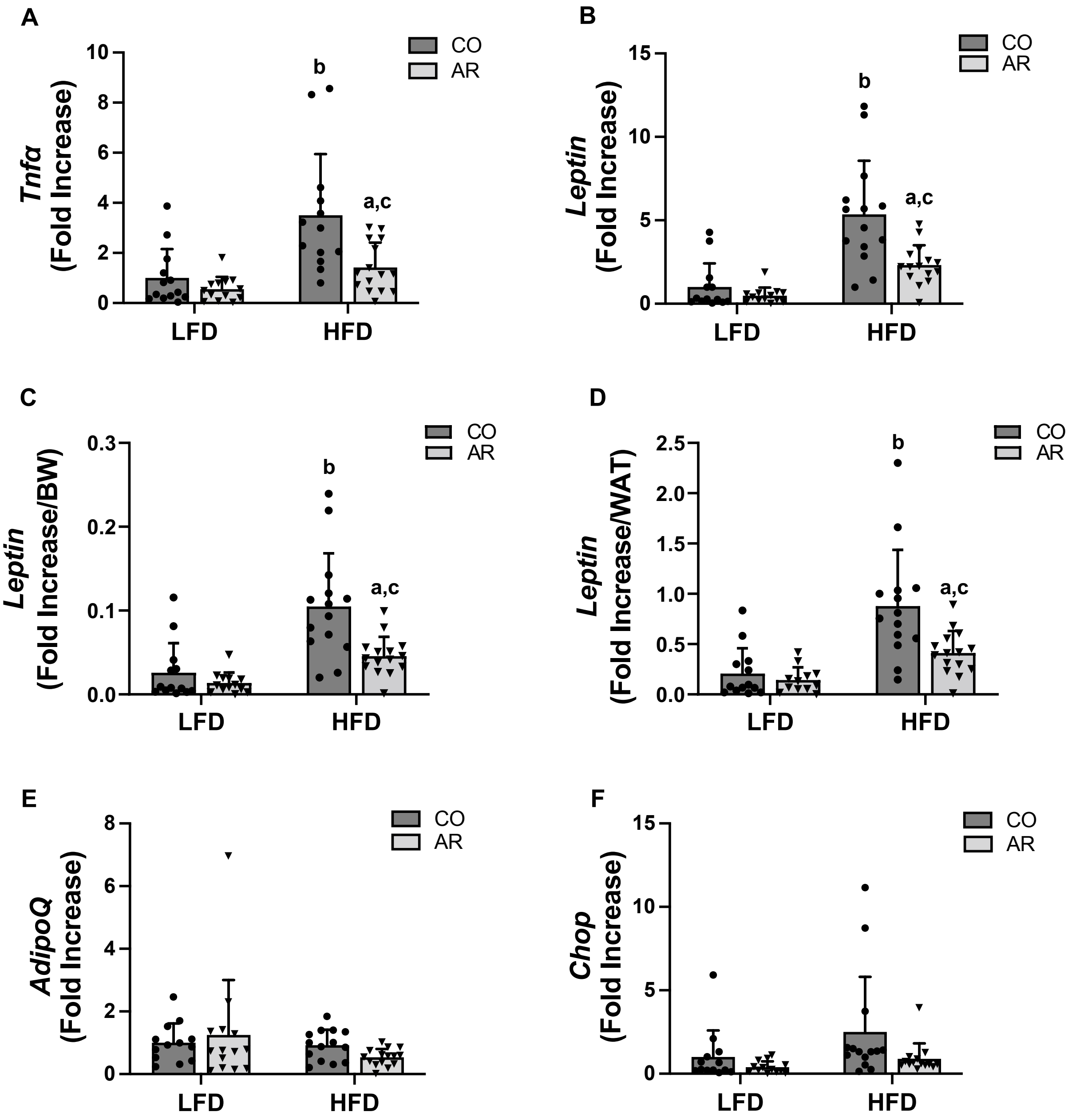

Supplemental Figure 5. Gene markers of inflammation, ER stress and metabolic hormones. Adipose mRNA levels for (A) *Tnfα* (B) *Leptin*, (C) *Leptin* normalized to body weight (BW), (D) *Leptin* normalized to white adipose tissue (WAT) weight, (E) *Adipoq* (Adiponectin) and (F) *Chop*

were measured by RT-PCR. Values are mean ± SD; $p < 0.05$, a - exposure effect, b - diet effect, c - interaction effect.

Supplemental Figure 6

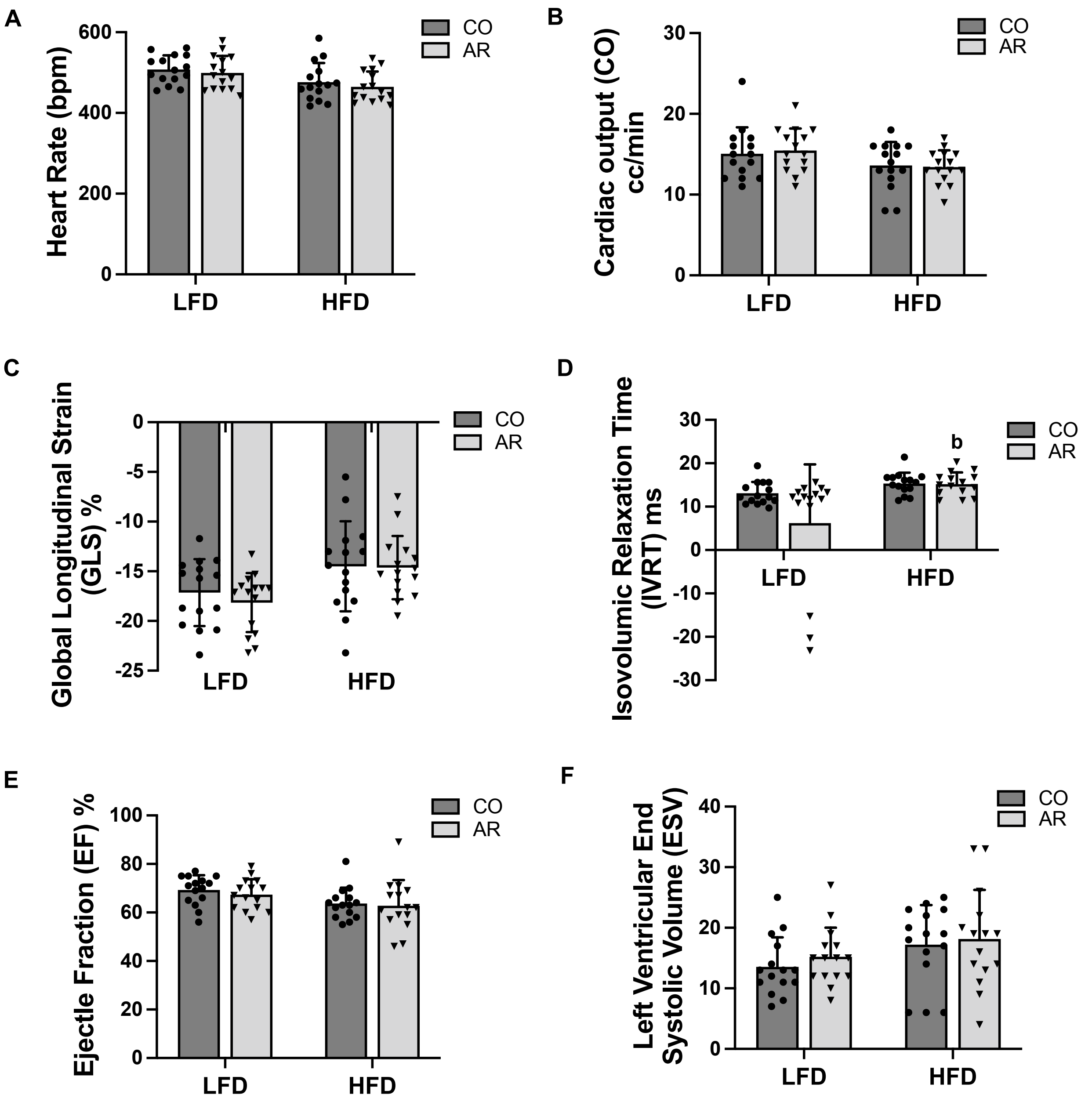

Supplemental Figure 6. Effects of Aroclor 1260 and HFD on cardiac function. Echocardiography was performed at week 23 of the study period. Measurements were made for (A) Heart rate (beats per minute), (B) Cardiac output, (C) Global longitudinal strain, (D) Isovolumic relaxation time, (E) Ejection fraction and (F) Left ventricular end systolic volume. Values are mean ± SD; p <0.05, a - Aroclor 1260 effect, b - diet effect, c - interaction effect.

Supplemental Figure 7

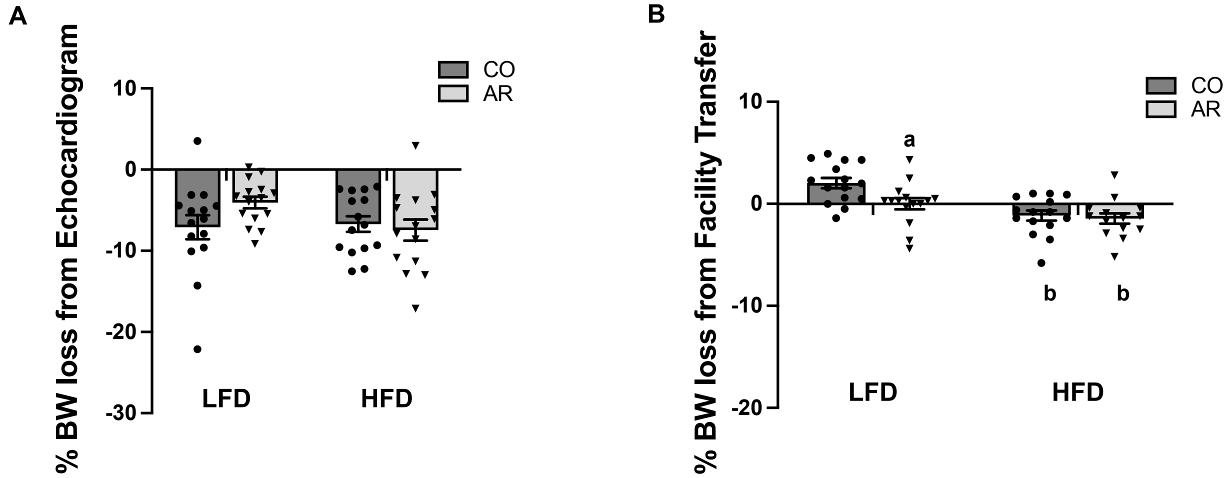

Supplemental Figure 7. Effects on body weight during the mid-study period. The percent loss in body weight (BW) after the (A) echocardiogram measurements and (B) transfer from primary animal facility to a satellite facility for echocardiogram measurements and back, were measured. No significant changes in body weight loss were noted between groups from the echocardiogram procedure but all experimental groups except for the LFD-CO group had body weight loss during this study period (week 23 - 26) before regaining it. This is also reflected in Supplemental Fig. 1A. Values are mean ± SD; p <0.05, a - Aroclor 1260 effect, b - diet effect, c - interaction effect.
